# Developmental modulation of schizophrenia risk gene methylation in offspring exhibiting cognitive deficits following maternal immune activation

**DOI:** 10.1038/s41380-025-03147-1

**Published:** 2025-08-29

**Authors:** Rebecca M. Woods, Harry G. Potter, Hager M. Kowash, Francesca McEwan, Isabella Harris, Joanna C. Neill, Christopher Murgatroyd, Jocelyn D. Glazier, Reinmar Hager

**Affiliations:** 1 Division of Evolution, Infection and Genomics, School of Biological Sciences, Manchester Academic Health Science Centre, Faculty of Biology, Medicine and Health, University of Manchester, Manchester, M13 9PL UK; 2 Department of Life Sciences, Centre for Biological Sciences, Manchester Metropolitan University, Manchester, M15 6BH UK; 3 Division of Neuroscience, School of Biological Sciences, Manchester Academic Health Science Centre, Faculty of Biology, Medicine and Health, University of Manchester, Manchester, M13 9PL UK; 4 Maternal and Fetal Health Research Centre, School of Medical Sciences, Manchester Academic Health Science Centre, Faculty of Biology, Medicine and Health, University of Manchester, Manchester, M13 9PL UK; 5 Division of Pharmacy & Optometry, School of Health Sciences, Manchester Academic Health Science Centre, Faculty of Biology, Medicine and Health, University of Manchester, Manchester, M13 9PL UK

**Keywords:** Neuroscience, Molecular biology, Genetics, Schizophrenia

## Abstract

Maternal infection during pregnancy has been shown in epidemiological studies to increase the risk of neurodevelopmental disorders, like schizophrenia, in the developing fetus. Epigenetic mechanisms are thought to play a crucial role in linking maternal immune activation (MIA) to a higher risk of schizophrenia in offspring by disrupting normal brain development. However, our knowledge of how these epigenetic mechanisms are altered and contribute to abnormal neurodevelopment remains limited. This lack of understanding has slowed progress in identifying therapeutic targets in particular for cognitive symptoms of neurodevelopmental disorders. Focusing on the cortex of offspring exposed to MIA who showed cognitive impairments, at both prenatal and postnatal stages, here we measured tissue concentrations of S-adenosylmethionine (SAM) and S-adenosylhomocysteine (SAH), using the SAM/SAH ratio as an indicator of overall methylation capacity. We also analyzed changes in the expression and activity of DNA methyltransferases (DNMTs), as well as DNA methylation (DNAm) patterns. Our findings revealed that MIA increased the SAM/SAH ratio and elevated both DNMT expression and activity in the fetal cortex. Surprisingly, these changes were not present after birth but resurfaced in adulthood, coinciding with cognitive deficits. These methylation pathway changes in adulthood were accompanied by altered DNAm patterns, with differentially methylated genes linked to schizophrenia risk and enriched in pathways related to neurodevelopment and neuronal signalling. These novel insights help establish a critical connection between MIA and epigenetic changes that contribute to cognitive deficits similar to those observed in schizophrenia.

## Introduction

### Maternal infection and schizophrenia risk

Numerous epidemiological studies support a link between maternal viral infection during pregnancy and increased risk of neurodevelopmental disorders (NDDs), including autism and schizophrenia, in the exposed fetus [1–5]. The prevailing hypothesis is that the maternal infection either directly or indirectly perturbs fetal brain development thereby predisposing these individuals to NDDs [2–7]. The mammalian brain develops over a protracted period, from early post-conception to adulthood, creating an extended window of vulnerability where neurodevelopmental processes can be altered by early-life environmental factors (e.g. maternal infection; [2, 8–10]). A central postulate is that maternal immune activation (MIA) in response to infection during pregnancy is influential in determining NDD risk [2–4, 11]. Despite growing support for a role of MIA in the aetiology of NDDs such as schizophrenia, our ability to study this in humans remains limited due to the protracted nature of disease aetiology, ethical considerations, and methodological challenges [12, 13]. To circumvent these constraints, rodent models of MIA are of growing importance to identify mechanisms and loci of dysfunction for potential new interventions [12–15].

We have characterised a MIA model using intraperitoneal (i.p.) administration of the viral mimetic polyinosinic:polycytidylic acid (poly(I:C)), on gestational day (GD) 15 in pregnant Wistar rat dams [16–20]. Using this model, we have recently demonstrated a cognitive deficit in adult offspring in the attentional set shifting task (ASST), which is predominantly linked to prefrontal cortex (PFC) function [19]. The ASST is considered analogous to the executive function deficits found in schizophrenia patients. Our subsequent research therefore aims to elucidate the mediating molecular and epigenetic mechanisms that promote the development of these schizophrenia-associated cognitive deficits following MIA [20, 21].

### Epigenetic mechanisms in schizophrenia and development

Epigenetic mechanisms are broadly defined as heritable mechanisms which regulate changes in gene function, but do not entail DNA sequence variations [22, 23]. Epigenetic mechanisms are highly relevant to neurodevelopment due to their ability to mediate interactions between environmental stressors and gene expression changes [24–26]. Altered epigenetic profiles have been reported in the brains of schizophrenia patients [27–29], with these schizophrenia-associated epigenetic patterns suggested to dysregulate genes involved in normal neurodevelopment [29–31].

DNA methylation (DNAm) is the most extensively studied epigenetic modification, most likely due to its stability which makes it amenable to downstream analysis [32]. DNAm occurs through addition of a methyl group to the 5th position of cytosine bases, commonly within cytosine-guanine dinucleotides (CpG sites). CpG sites are often clustered within CpG islands (CGIs) near gene promoters, methylation of which represses gene expression [33–35]. DNAm patterns are highly development- and tissue-specific, with the brain showing overall hypermethylation relative to other tissues [36, 37].

Establishment of mammalian DNAm occurs through the activities of DNA methyltransferase (DNMT) enzymes. DNMTs catalyse the transfer of the methyl group, from the methyl-donor, S-adenosyl-methionine (SAM), onto cytosine residues, producing S-adenosyl-homocysteine (SAH; [38]). SAH, in turn, inhibits DNMT activity in a negative feedback loop. Accordingly, an increased SAM/SAH ratio is indicative of increased cellular methylation capacity [39]. DNMTs are comprised of de novo DNMTs (DNMT3a/DNMT3b), which establish methylation patterns in previously non-methylated DNA, and maintenance DNMTs (DNMT1), which accurately copy DNAm patterns during cellular divisions [38]. DNMTs are critical for forming correct DNAm patterns, thereby transcriptionally programming cell fate and differentiation decisions during neurodevelopmental processes, particularly neurogenesis and gliogenesis [40–42]. Further, dynamic DNAm remodelling occurs during postnatal neurodevelopment in line with ongoing synaptic plasticity, learning and memory formation [41, 43].

### Epigenetic mechanisms and MIA

Perturbations to normal epigenetic processes are predicted to result in altered neurodevelopmental trajectories and predisposition to disease [41, 44, 45]. Hence, epigenetic mechanisms, particularly DNAm, are proposed as key mediators between MIA and offspring schizophrenia risk [21, 24, 46, 47]. In support of this, it has been demonstrated that inflammatory signalling pathways can induce DNMT activity and thereby alter DNAm [48–50]. We hypothesised that poly(I:C)-induced MIA causes functional changes in DNMT expression/activity, leading to altered methylation capacity and thus developmental changes in genomic DNAm in the offspring PFC. We predicted that these changes are functionally relevant in offspring that exhibit cognitive deficits, reminiscent of those seen in schizophrenia. To address this hypothesis, we investigated how MIA-elicited responses influence cellular methylation pathways and DNAm patterns across a developmental timeline in the cortices of fetuses and offspring of poly(I:C)-treated dams.

## Materials and methods

### Animal procedures

All animals used in this study were from our previously published cohorts ([17, 19]; see Supplement S1 for full details), and all experiments were conducted in accordance with the Animals in Scientific Procedures Act (ASPA) 1986. All details of the study design are provided following the MIA Model Reporting Guidelines Checklist devised by Kentner et al. ([51]; Supplement S2), and a study timeline is provided in Fig. S1.1.

Briefly, adult Wistar rats (Charles River Laboratories, UK) were maintained in the Biological Services Facility at the University of Manchester under standard conditions. On GD15, time-mated dams were pseudo-randomly assigned to receive a single i.p. injection of 10 mg/kg bodyweight of low molecular weight poly(I:C) (InvivoGen, London, UK; N = 15) or saline only (vehicle control; N = 15), between 08:00–10:00 to minimise circadian influence on immune outcomes [52]. 3 h post-injection dam tail vein plasma was collected for analysis of pro-inflammatory cytokines IL-6 and TNFα, to confirm an acute MIA response (Fig. S1.2). All dams were subsequently randomly assigned to either gestational (N = 12) or postnatal (N = 18) cohorts. For dams assigned to gestational cohorts the fetuses were harvested and sexed as described previously [17], and fetal whole brains isolated. Dams assigned to postnatal cohorts birthed naturally and on postnatal day (PD) 1 pups were sexed [19, 53] and litters culled to 10, with equal sex ratios maintained where possible [19, 54]. The remaining pups were then assigned to be culled on PD21, PD35 and PD175 for brain sample collection, with behavioural testing performed at PD35 and PD120-140 as described previously [19]. A summary of all animals used in this study is provided in Table S1.1.

### Preparation of isolated brains for downstream analysis

Collected whole brains were bisected into left and right hemispheres for nucleic acid or protein isolation, respectively. Dissections were performed as directed by the Rat Brain Stereotaxic Co-ordinates ([55]; Supplement S1): for GD21, when the PFC has not yet formed [56], the frontal cortex (FC) only was dissected, whereas for PD21, PD35 and PD175, the PFC was dissected. We selected the PFC for its involvement in cognition, particularly its role in the ASST [57, 58] in which these MIA-offspring displayed a deficit [19].

### Nucleic acid extraction and quantification

DNA and RNA were extracted from cortical tissue samples using the DNeasy Blood and Tissue kit (Qiagen, Manchester, UK) and RNeasy Plus Mini Kit (Qiagen, Manchester, UK), respectively, according to the manufacturer’s guidelines. RNA and DNA concentration was measured using the ThermoFisher NanoDrop® (Waltham, USA). For sequencing-based assays, intact DNA concentration was determined using the QuantiFluor® ONE dsDNA System (Promega, Southampton, UK) according to the manufacturer’s guidelines.

### Real-time quantitative PCR (qPCR)

RNA was reverse transcribed using the QuantiTect Reverse Transcription Kit (Qiagen, Manchester, UK) according to the manufacturer’s guidelines. We quantified relative gene expression by qPCR using Qiagen QuantiTect Primers for candidate genes of interest (*Dnmt1, Dnmt3a, Dnmt3b*; Table S1.2), and QuantiFast SYBR Green RT-PCR Kit (Qiagen, Manchester, UK) following the manufacturer’s instructions. Candidate gene expression was normalised to the geometric mean of three reference genes (*Gapdh, Ubc* and *Mdh1;* PrimerDesign Ltd., Chandler’s Ford, UK), which were determined to be stable in these samples based on GeNorm analysis ([59]; Supplement S1).

### Isolation of nuclear and cytosolic cellular fractions

Protein lysates were prepared from cortical tissue samples using the Nuclear Extract kit (Active Motif, Waterloo, Belgium) following the kit specifications to isolate cytosolic and total nuclear protein fractions. Protein concentration of the individual fractions was quantified using a Bradford Protein Assay (Bio-Rad, Hertfordshire, UK), according to the manufacturer’s instructions.

### DNMT activity assay

The Epiquick DNMT Activity/Inhibition Assay Ultra kit (Epigentek, Farmingdale, USA) was used to quantify total nuclear DNMT activity (comprising both de novo and maintenance DNMT activity) from total nuclear fractions (10 μg protein). Assays were performed following the manufacturer’s instructions, and absorbance read on a Biotek Synergy H1 microplate reader (Agilent, Cheadle, UK) at 450 nm primary wavelength and 655 nm reference wavelength. Total DNMT activity was calculated according to the manufacturer’s instructions.

### Quantification of cytosolic SAM and SAH

One carbon (1C) metabolites, SAM and SAH were quantified in cytosolic fractions (250 μg protein) using ELISAs: SAM ELISA Kit (Aviva Systems Biology, San Diego, USA) and SAH ELISA (Cell Biolabs, San Diego, USA), respectively. Absorbance at both primary (450 nm) and reference (570 nm for SAM; 620 nm for SAH) wavelengths were read on a Biotek synergy H1 plate reader (Agilent, Cheadle, UK). Sample SAM and SAH concentrations were calculated according to the manufacturer’s instructions.

### Total genomic DNA methylation ELISA

An initial, rapid estimation of total genomic DNAm was performed using a %5mC ELISA kit (ENZO, Exeter, UK) following the manufacturer’s instructions. Endpoint absorbance was read on a FLUOstar Omega plate reader (BMG Labtech, Aylesbury, UK) at 410 nm. Sample %5mC DNA values were interpolated from the *E. Coli* DNA standard curve, with correction for rat CpG density per genome length, as identified by Su et al. [60].

### Reduced representation bisulphite sequencing (RRBS)

PFC DNA from behaviourally tested [19] adult females (n = 4 vehicle and poly(I:C)) was used for RRBS to identify gene-specific DNAm changes. As we sought to associate epigenetic changes with behavioural deficits, adult females were selected as they had demonstrated executive function deficits in the ASST, analogous to those observed in schizophrenia patients ([19]; Supplement S1). Adult male offspring did not engage with the ASST and we were unable to determine the presentation of a cognitive deficit. Therefore, males were excluded from RRBS analysis (Supplement S1).

RRBS library preparation and sequencing was performed by Diagenode using the Premium Reduced Representation Bisulphite Sequencing Kit (Diagenode, Liège, Belgium) following the manufacturer’s instructions and published workflow ([61]; Supplement S1).

### Statistical analysis and bioinformatics

- Power analysis
  We calculated sample size using G*Power (v3.1.9.2. Düsseldorf, Germany). A sensitivity analysis, assuming a medium-large effect size (f = 0.25–0.4) was based on previous estimates from our and other molecular studies in MIA models [17–20, 62], showed a minimum of 5–6 offspring/sex/group/age was required for molecular analysis to be sufficiently powered (1-β = 0.8), with a type I error rate (α) = 0.05.
- Statistical analysis of molecular data
  Statistical analysis of fetal/offspring molecular outputs (excluding RRBS) were performed using SPSS v28.0 (IBM). Between-group analysis of molecular outputs used a general linear mixed model (GLMM), including dam as a random factor and the following predictors: fixed factors (sex, group) and co-variates (maternal IL-6 and TNFα), with p-values ≤ 0.05 considered statistically significant and 0.05 < p ≤ 0.075 highlighted as approaching significance. For all GLMMs, where sex or group*sex interactions were significant/approaching significance, a GLMM was performed within a single sex with group as a fixed factor and maternal cytokines as co-variates. Data are presented as mean ± SEM with numbers of dams per group (N) and fetuses/offspring per sex per group (n) indicated. Sex*group interactions are not indicated on Figures - only within-sex analyses are shown. Where appropriate, correlations were used to evaluate the relationship between quantitative measures: when both variables were normally distributed, Pearson’s correlations were used; where one or more variables were non-normally distributed, Spearman’s (rho) correlations were used.
- Analysis of RRBS data
  Standard bioinformatics were performed by Diagenode (Liège, Belgium; Supplement S1), including quality control checks and sequence read alignment to the Rn5.0 reference genome [63–65]. A pairwise comparison between ‘Poly(I:C)’ versus ‘Vehicle’ was used to identify differentially methylated CpGs (DMCs) and regions (DMRs), the latter comprising 1000 bp sequence stretches (Supplement S3). Logistic regression was used to compare methylation between groups at each DMC/DMR, with the sliding window model to correct p-values to q-values (Supplement S3). DMCs/DMRs were filtered using a q-value cut-off ≤0.01 and a methylation difference ≥25%, accounting for false discovery of DMCs/DMRs due to technical variability (e.g., coverage, read depth) and inherent heteroscedasticity of raw methylation values [61, 66, 67]. Filtered DMCs/DMRs were annotated to genes using annotatr (R/Bioconductor; [68]), employing refGene and CpG annotations from Rn5.0 [69]. The annotation comprised two categories: (i) CpG context annotation (Supplement S4): including overlapping a known CGI; ≤2000 bp flanking region of a CGI (CpG shore); ≤2000bp of a CpG shore (CpG shelves) or outside these regions (open sea); (ii) gene context annotation (Supplement S5): including within an exon, intron or promoter, or not mapped to a gene (intergenic).
  The list of differentially methylated genes (i.e. genes to which ≥1 filtered DMC/DMR was annotated), hereafter referred to as the RRBS gene list, was used for gene function analysis. We analzyed Gene ontology (Supplement S6) using topGO (R/Bioconductor; [70]), and pathway enrichment analysis conducted using Kyoto Encyclopaedia of Genes and Genomes (KEGG) terms within ReactomePA (R/Bioconductor; [71]; Supplement S6). Finally, to assess the validity of the RRBS gene list for schizophrenia research, schizophrenia risk gene lists were collated from published genetic [72, 73], expression [72, 74] and epigenetic studies [29, 72] in schizophrenia. All gene identifiers were converted to rat orthologues using g:Profiler [75] and a Fisher’s exact test used to assess enrichment of the RRBS gene list against each of compiled gene list.

## Results

### MIA induces increased methylation capacity and global DNAm in the fetal cortex

**Fig. 1:**
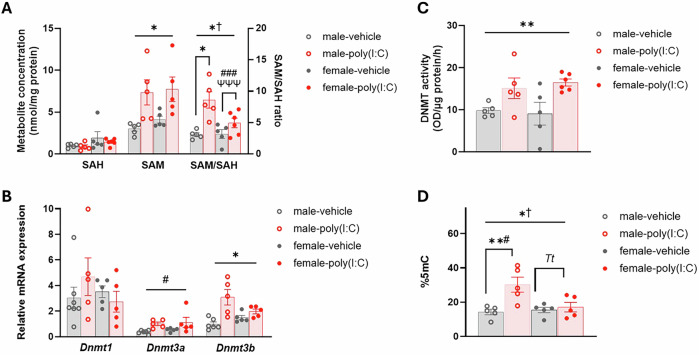
Methylation pathway and %5mC changes in the GD21 FC of fetuses from poly(I:C)-treated dams. **A** Cytosolic SAH and SAM concentration and SAM/SAH ratio. **B** *Dnmt* mRNA expression relative to the geometric mean of three housekeeping genes (see Methods). **C** Total nuclear DNMT activity. **D** Global DNAm measured as %5mC. Bars represent mean ± SEM (N = 5–6; n = 5–7). ― shaped bars show GLMM results across the analysis they overlap, Π-shaped bars represent post-hoc GLMM results within a single sex. Symbols show main effects of: sex, ^†^p ≤ 0.05; group, ^*t*^0.05 < p < 0.075, *p < 0.05, **p < 0.01; maternal IL-6, ^#^p < 0.05, ^###^p < 0.001; maternal TNFα, ^*T*^0.05<p<0.075, ^ψψψ^p < 0.001.

While there were no differences in cytosolic SAH concentration (Fig. 1A), treatment group affected cytosolic SAM concentration (GLMM: F_1,9.08_ = 8.87, p = 0.015) and, consequently, SAM/SAH ratio (GLMM: F_1,10_ = 7.98, p = 0.018), with both significantly higher in the GD21 FC of poly(I:C)-fetuses compared to vehicle-fetuses (Fig. 1A). Sex also influenced SAM/SAH ratio (GLMM: F_1,10_ = 5.72, p = 0.038; Fig. 1A), with higher ratios in the male GD21 cortex than in the female cortex, alongside a group*sex interaction (GLMM: F_1,10_ = 11.35, p = 0.007). Subsequent analysis by sex revealed that in females both maternal IL-6 (GLMM F_1,4_ = 95.24, p < 0.001) and TNFα (GLMM: F_1,4_ = 79.47, p < 0.001) influenced SAM/SAH ratio, corresponding to positive correlations between cytosolic SAM/SAH ratio and maternal IL-6 (*rho* = 0.980, p < 0.001), and TNFα (*rho* = 0.976, p < 0.001). In males, treatment group primarily affected SAM/SAH ratio (GLMM: F_1,5_ = 12.34, p = 0.017), with increased SAM/SAH ratio in the GD21 cortex of poly(I:C)-males relative to vehicle-males.

*Dnmt* mRNA expression (Fig. 1B) was also altered in response to MIA. *Dnmt3a* expression was predicted by maternal IL-6 (GLMM: F_1,11_ = 9.80, p = 0.011), corresponding to a positive correlation between maternal IL-6 (*rho* = 0.617, p = 0.006) and *Dnmt3a* expression in the GD21 cortex. *Dnmt3b* expression was affected by treatment group (GLMM: F_1,8.55_ = 8.16, p = 0.020) with elevated *Dnmt3b* expression in the GD21 cortex of poly(I:C)-fetuses compared to vehicle-fetuses. There were no significant differences in *Dnmt1* expression. The increased *Dnmt3a/b* mRNA expression in the GD21 cortex corresponded to increased nuclear DNMT activity (Fig. 1C) in poly(I:C)-fetuses relative to vehicle-fetuses (main effect of group; GLMM: F_1,12_ = 15.22, p = 0.002).

Our analysis of total global DNAm (%5mC) in the GD21 cortex (Fig. 1D) showed a group*sex interaction (GLMM: F_1,16_ = 6.42, p = 0.047), alongside main effects of both sex (GLMM: F_1,16_ = 4.49, p = 0.05) and group (GLMM: F_1,16_ = 9.73, p = 0.007). Subsequent analysis by sex revealed main effects of treatment group (GLMM: F_1,4_ = 34.61, p = 0.004) and maternal IL-6 (GLMM: F_1,4_ = 8.35, p = 0.045) in male-fetuses, while in female-fetuses there were trends to main effects of maternal TNFα (GLMM: F_1,3_ = 8.28, p = 0.064) and treatment group (GLMM: F_1,3_ = 7.51, p = 0.071).

### Methylation capacity and total DNAm in the juvenile and adolescent PFC is influenced primarily by sex

Given the observed MIA-induced changes in methylation capacity and DNAm in the fetal FC, we sought to determine if such changes persist postnatally into the juvenile (PD21) and adolescence (PD35) periods.

**Fig. 2:**
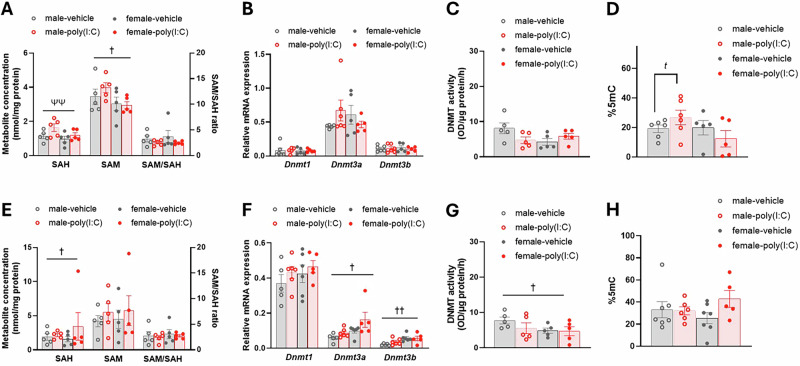
Methylation pathway and %5mC changes in the PD21 and PD35 PFC of offspring from poly(I:C)-treated dams. **A** PD21 PFC cytosolic SAH and SAM concentration and SAM/SAH ratio. **B** PD21 PFC *Dnmt* mRNA expression relative to the geometric mean of three housekeeping genes (see Methods). **C** PD21 PFC total nuclear DNMT activity. **D** PD21 PFC global DNAm measured as %5mC. **E** PD35 PFC cytosolic SAH and SAM concentration and SAM/SAH ratio. **F** PD35 PFC *Dnmt* mRNA expression relative to the geometric mean of three housekeeping genes (see Methods). **G** PD35 PFC total nuclear DNMT activity. **H** PD35 PFC global DNAm measured as %5 mC. Bars represent mean ± SEM (N = 5–6; n = 5–7). ― shaped bars show GLMM results across the analysis they overlap, Π-shaped bars represent post-hoc GLMM results within a single sex. Symbols show main effects of: sex, ^†^p ≤ 0.05, ^††^p < 0.01; group, ^*t*^0.05<p < 0.075; maternal TNFα, ^ψψ^p < 0.01.

In the PD21 PFC, maternal TNFα affected SAH concentration (GLMM: F_1,18_ = 9.57, p = 0.006; Fig. 2A), with a positive correlation between maternal TNFα and cytosolic SAH (r = 0.589, p = 0.006), while SAM concentration was influenced by sex (GLMM: F_1,18_ = 4.46, p = 0.049; Fig. 2A) with elevated cytosolic SAM in males relative to females. However, these did not correspond to any differences in SAM/SAH ratio (Fig. 2A), *Dnmt* expression (Fig. 2B) or nuclear DNMT activity (Fig. 2C) in the PD21 PFC. Analysis of global DNAm (%5mC) in the PD21 PFC demonstrated a group*sex interaction (GLMM: F_3,15_ = 4.06, p = 0.027). Subsequent analysis by sex revealed a trend to a main effect of treatment group in the males (GLMM: F_1,9_ = 4.28, p = 0.069; Fig. 2D), with elevated %5mC in poly(I:C)-males relative to vehicle-males, but no significant differences in %5mC in the female PD21 PFC.

In PD35 PFC, there were no effects of treatment group or maternal cytokines on 1C metabolite availability, DNMT mRNA expression/activity, or %5mC (Fig. 2E–H). However, cytosolic SAH concentration was influenced by sex (GLMM: F_1,3.48_ = 16.83, p = 0.020), with females having higher cytosolic SAH concentrations than males (Fig. 2E), although there were no corresponding changes in cytosolic SAM concentration or SAM/SAH ratio (Fig. 2E). Sex also influenced both *Dnmt3a* (GLMM: F_1,19_ = 5.25, p = 0.05) and *Dnmt3b* expression (GLMM: F_1,7.06_ = 15.88, p = 0.005) in the PD35 PFC, with higher expression of both enzymes in females compared to males (Fig. 2F). However, this corresponded surprisingly with reduced nuclear DNMT activity in the PD35 PFC of females relative to males (main effect of sex; GLMM: F_1,11.42_ = 5.37, p = 0.040; Fig. 2G), but there was no effect of sex on %5mC (Fig. 2H).

### MIA induces minor changes in methylation capacity in the adult PFC but with no effect on overall quantity of global DNAm

We next determined if any changes in these methylation pathways occur in the PFC during the emergence of the observed cognitive deficit in adulthood (PD175; [19]).

**Fig. 3:**
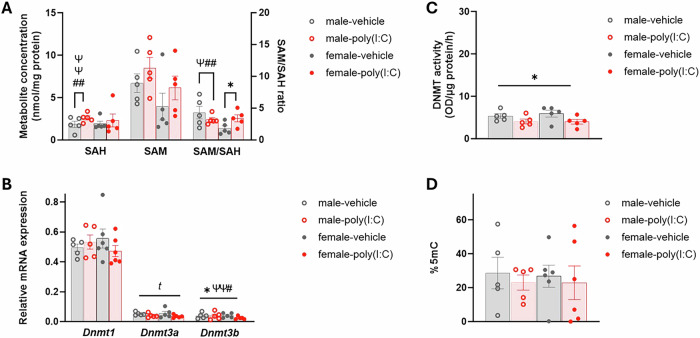
Methylation pathway and %5mC changes in the PD175 PFC of offspring from poly(I:C)-treated dams. **A** Cytosolic SAH and SAM concentration and SAM/SAH ratio. **B** *Dnmt* mRNA expression relative to the geometric mean of three housekeeping genes (see Methods). **C** Total nuclear DNMT activity. **D** Global DNAm measured as %5 mC. Bars represent mean ± SEM (N = 5–6; n = 5–7). Significance bars show significant GLMM results, ― shaped bars show results across the analysis they overlap, Π-shaped bars represent post-hoc results within a single sex. Symbols show main effects of: group, ^*t*^0.05<p < 0.075, *p < 0.05; maternal IL-6, ^#^p < 0.05, ^##^p < 0.01; maternal TNFα, ^ψ^p < 0.05, ^ψψ^p < 0.01.

For cytosolic SAH concentration (Fig. 3A), there was a trend to a group*sex interaction (GLMM: F_1,4.72_ = 4.84, p = 0.072) and subsequent analysis by sex showed main effects of maternal IL-6 (GLMM: F_1,6_ = 25.23, p = 0.002) and TNFα (GLMM: F_1,6_ = 19.46, p = 0.005) on cytosolic SAH in the male PD175 PFC, and no significant differences in the females. Further, while there were no differences in cytosolic SAM concentration in the PD175 PFC, there was a trend to a group*sex interaction (GLMM F_3,14_ = 2.88, p = 0.073) on SAM/SAH ratio (Fig. 3A). Subsequent analysis by sex showed a treatment group effect in females (GLMM: F_1,7_ = 10.20, p = 0.015) with increased SAM/SAH ratio in PD175 PFC of poly(I:C)-females relative to vehicle-females, while in the males there were main effects of maternal TNFα (GLMM: F_1,6_ = 13.35, p = 0.011) and maternal IL-6 (GLMM: F_1,6_ = 14.38, p = 0.009) on SAM/SAH ratio, although these did not demonstrate any significant correlations.

There were also disturbances in *Dnmt* mRNA expression in the PD175 PFC (Fig. 3B), with a trend to a main effect of group on *Dnmt3a* expression (GLMM: F_1,18_ = 4.26, p = 0.054), with reduced *Dnmt3a* expression in poly(I:C)-offspring compared to vehicle-offspring. Likewise, *Dnmt3b* expression was also affected by treatment group (GLMM: F_1,13_ = 8.42, p = 0.012), with reduced *Dnmt3b* expression in poly(I:C)-offspring relative to vehicle-offspring, alongside effects related to maternal TNFα (GLMM: F_1,13_ = 9.27, p = 0.009) and maternal IL-6 (GLMM: F_1,13_ = 6.19, p = 0.027), although no cytokine correlations were significant. There were no differences in *Dnmt1* expression (Fig. 3B). In concordance with reduced *Dnmt3a/3b* mRNA expression, nuclear DNMT activity was also affected by treatment group (GLMM: F_1,18_ = 7.26, p = 0.015; Fig. 3C), with reduced DNMT activity in the PD175 PFC of poly(I:C)-offspring relative to vehicle-offspring. However, despite this collective evidence for disturbed methylation capacity and DNMT activity in response to MIA, there were no differences in %5mC in the PD175 PFC (Fig. 3D).

### MIA induces gene-specific DNAm changes in the adult PFC

**Fig. 4:**
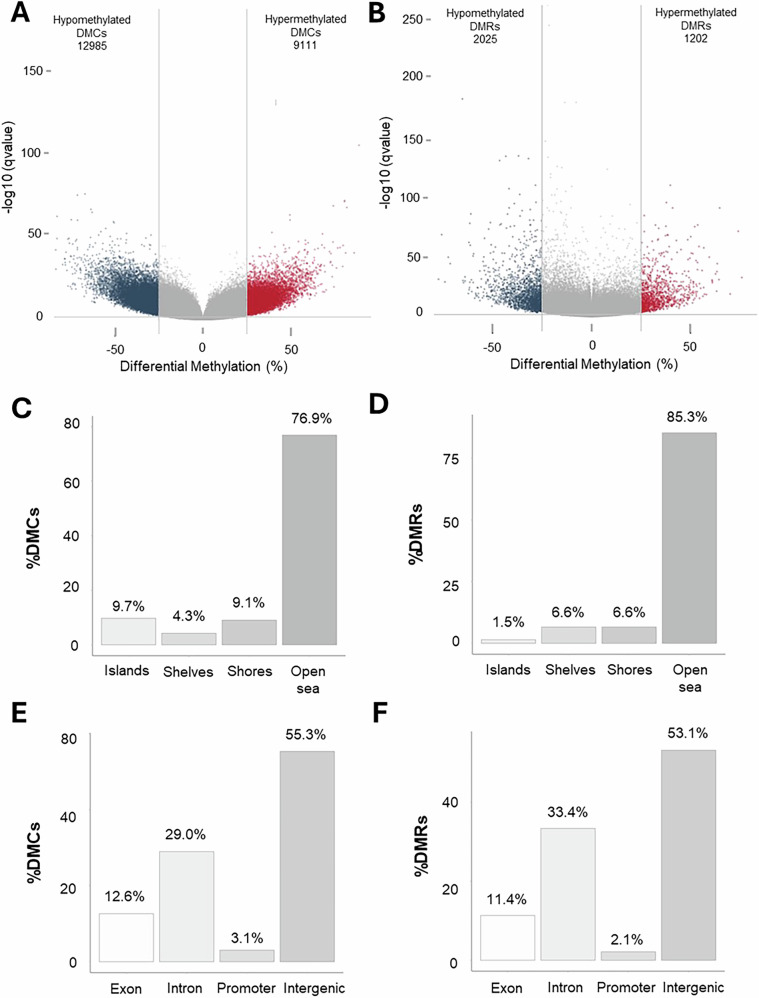
Identification of differentially methylated cytosines (DMC) and regions (DMR). Volcano plots show the number of **A**. DMCs and **B**. DMRs with changed patterns of methylation between “Poly(I:C)” and “Vehicle” with ± 25% difference (demarcated by the grey lines) and a q-value ≤ 0.01. The difference in methylation (%) is given on the x-axis while the y-axis represents the significance (q-value) of the difference. Bar charts depict CpG and gene context annotation of DMCs and DMRs which surpass the ± 25% methylation difference and q-value ≤ 0.01 cut off. **C** DMC mapping to CpG islands. **D** DMC mappings to genic location. **E** DMR mapping to CpG islands. **F** DMR mapping to genic location.

A key aim of this study was to link epigenetic changes with MIA-associated behavioural deficits. Previous work showed that MIA-induced cognitive deficits occurred in these animals between PD35-175 [19]. While the global DNAm (%5mC) assay did not identify any difference in DNAm in the adult PFC in response to MIA, the possibility that there were MIA-induced gene-specific DNAm changes cannot be excluded, especially as DNMT activity was significantly reduced. To examine gene-specific methylation changes in the adult PFC, which might be associated with behavioural changes, we utilised RRBS analysis of DNA extracted from MIA-exposed adult females that had demonstrated a cognitive deficit in the ASST [19], a task dependent on PFC-mediated executive function. As adult male offspring did not engage with the ASST we were unable to determine the presentation of a similar deficit [19], thus they were excluded from the RRBS analysis. Using the cut-off of q-value ≤ 0.01 and methylation difference ≥25%, we identified 22,096 DMCs and 3227 DMRs between poly(I:C) and vehicle groups (Supplement S3). These filtered DMCs/DMRs were used as the focus of the study. Of these filtered DMCs, 12,985 (58.8%) were hypomethylated and 9111 (41.2%) were hypermethylated (Fig. 4A), while for the DMRs 2025 (62.7%) were hypomethylated and 1202 (37.3%) were hypermethylated (Fig. 4B).

CpG (Supplement S4) and gene (Supplement S5) context annotation showed that the majority of the filtered DMCs (76.9%) mapped to open sea regions (Fig. 4C), with most mapped to intergenic regions (55.3%; Fig. 4E), and a relatively low proportion mapped to CGIs (9.7%), shores (9.1%) and shelves (4.3%; Fig. 4C). Likewise, for filtered DMRs, most mapped to open sea regions (85.3%; Fig. 4D), with most again located in intergenic regions (53.1%; Fig. 4F) and the lowest proportion mapped to CGIs (1.5%) with identical proportions (6.6%) mapped to shores and shelves (Fig. 4D). Only 3.1% and 2.1% of DMCs and DMRs mapped to promoters, while 12.6% and 11.4% mapped to exons, and 29.0% and 33.4% to introns, respectively (Fig. 4E, F).

These genic annotations of DMC/DMRs were used to determine their gene identity. A total of 4030 differentially methylated genes were identified (Supplement S5).

### MIA-induced differential methylation occurs at genes involved in brain development and function

**Fig. 5:**
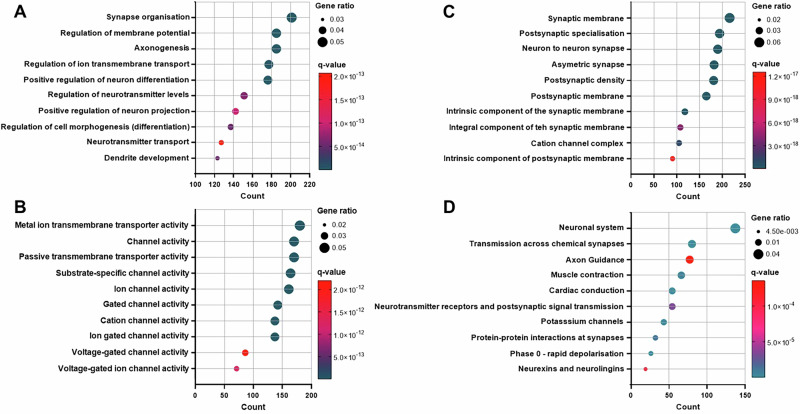
Gene Ontology and KEGG pathway analysis of MIA-induced differentially methylated genes. **A** Gene Otology: Biological processes. **B** Gene Ontology: Molecular functions. **C** Gene Ontology: Cellular components. **D** KEGG pathway. Y-axis shows the top ten processes/pathways, x-axis indicates the total count of differentially methylated genes enriched for those processes/pathways. Circle size represents the gene ratio (calculated as number of genes enriched/total number of genes in the dataset), while significance (q-value) is indicated by colour, with red being the most significant. All analyses used a hypergeometric model with false discovery rate to correct p-values to q-values.

To determine the function of the MIA-associated differentially methylated genes, we performed Gene Ontology analysis, inclusive of biological process, molecular function and cell component (Supplement S6). The top ten biological processes (Fig. 5A) demonstrated an enrichment for functions in neuronal development, including synapse/axon generation and cell differentiation, aligning with the top ten molecular functions, which incorporated ion transmembrane transport and channel activity (Fig. 5B). Concordant with these observations, the top ten cell components were primarily enriched for synaptic membranes (Fig. 5C). Taken together, these data suggest MIA induces differential methylation of genes involved in neuronal function and signalling, with the potential to impact on pathways involved in neurodevelopmental processes. We additionally performed a KEGG pathway analysis (Supplement S6), which showed that the top ten enriched pathways were those involved in normal neuronal functional and neuronal signalling pathways (Fig. 5D).

Collectively, these results provide evidence that MIA is associated with altered regulation of genes involved in neurodevelopmental processes and neuronal signalling in the offspring through modified DNAm.

### MIA-induced differential methylation occurs at genes associated with schizophrenia

**Fig. 6:**
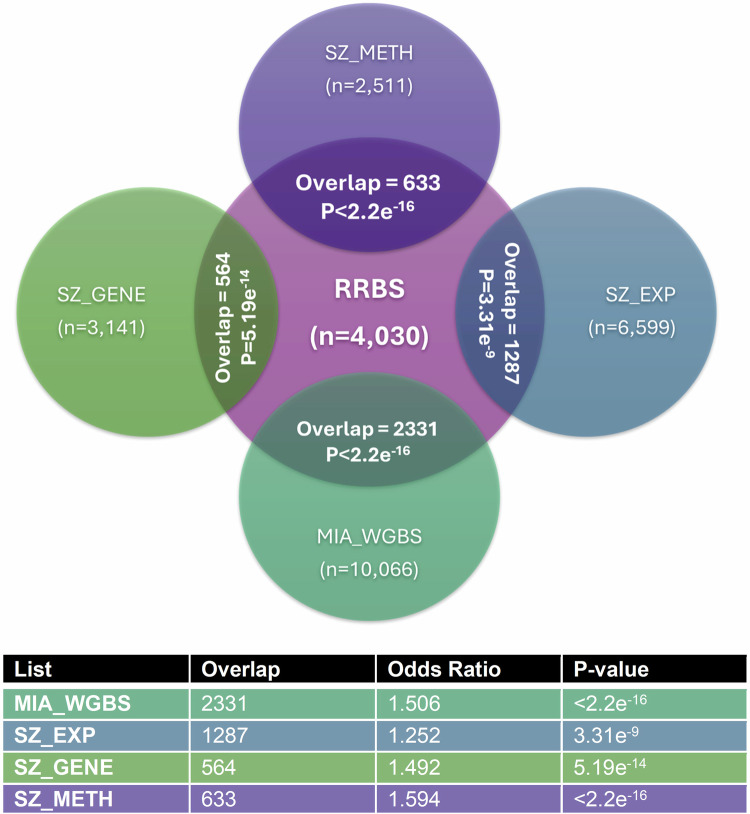
RRBS gene list overlaps with schizophrenia risk genes. Figure demonstrates the overlap between the differentially methylated genes identified by the RRBS analysis in this study to other gene datasets (refer to Supplementary Methods Section 1.4) including: schizophrenia variant genes (SZ_GENE), genes differentially methylated in schizophrenia (SZ_METH), genes differentially expressed in schizophrenia (SZ_EXP), and genes differentially methylated in the PFC of male mice exposed to MIA (MIA_WGBS) see Table S1.3 for full details. A Fisher’s Exact test was used to compare the significance of the overlaps as indicated in the Table.

Further analyses compared the RRBS gene list to compiled schizophrenia gene lists (Supplement S1), comprising genes with known genetic variants (SZ_GENE), genes differentially methylated (SZ_METH) and genes differentially expressed (SZ_EXP) in schizophrenia. There was a significant overlap between our dataset and all compiled schizophrenia gene lists (Fig. 6). The RRBS gene list was also compared to differentially methylated genes identified from a MIA mouse model [62], which showed a highly significant (>50%) overlap with 2331 genes differentially methylated in both studies (Fig. 6). Taken together, these data provide strong support for the notion that differential methylation of schizophrenia risk genes in response to MIA contribute to the aetiology of the MIA-induced behavioural deficits observed.

## Discussion

This study investigated the hypothesis that MIA, induced by the viral memetic poly(I:C), perturbs neuro-epigenetic patterns in the fetal brain, leading to dysregulated neurodevelopment and altered neuronal function, thereby predisposing offspring to schizophrenia-like behavioural abnormalities. Our results provide evidence of MIA-induced increases in SAM/SAH ratio, with concomitant increases in de novo DNMT expression/activity and DNAm in the fetal cortex. These MIA-induced changes in cortical methylation pathways appear to be absent postnatally, and no changes were observed in the juvenile-adolescent period. However, in adulthood we detected altered methylation patterns were apparent, with elevated SAM/SAH ratios alongside reduced DNMT expression/activity. Further, the adult PFC showed significant MIA-associated differential methylation of genes highly enriched for neurodevelopmental pathways and known to influence schizophrenia risk.

### MIA induces acute changes to fetal cortical methylation pathways and total DNAm quantity

As expected, our study confirmed higher tissue cytosolic SAM than SAH concentration, with good concordance to previous SAM/SAH ratios reported in rodent brains [38, 75–77]. SAM forms SAH after the donation of its methyl group for cellular methylation processes, which is crucial for establishing normal DNAm patterns [78]. Therefore, the SAM/SAH ratio is indicative of cellular methylation capacity [38, 39]. Here, we show that regulation of this metabolic cycle is disrupted in the fetal cortex following MIA, leading to increased cytosolic SAM concentration and elevated SAM/SAH ratio. Interestingly, our previous studies have shown that placental transport of leucine, primarily transported by system-L amino acid transport (which also transports methionine [79]), is increased at GD21 following poly(I:C)-induced MIA [19]. As methionine is the precursor for SAM production, an increased transplacental flux of methionine would be consistent with the data presented here in supporting increased GD21 methyl demand. Such changes in methylation capacity have been shown to induce altered DNAm patterns and are associated with behavioural phenotypes relevant to NDDs [75, 77, 80]. Likewise, increased prenatal SAM supplementation has been shown to induce DNAm changes at genes involved in neuroinflammation and neurodevelopment [81], aligning with observations made here.

Prenatal neurodevelopment involves extensive cellular differentiation/proliferation, processes influenced by significant DNAm changes, driven by DNMT activity [38]. Accordingly, the expression of all three DNMT enzymes was found to be high in the fetal cortex, with selective MIA-induced increases in de novo DNMT expression. We found GD21 *Dnmt3a* expression was positively correlated with maternal plasma IL-6 concentration. Previous studies have shown that MIA induces fetal neuroinflammatory disturbances, particularly elevated IL-6, IL-1β, TNFα and IL-10 [10, 21, 82], and these cytokine signalling pathways induce increased DNMT expression/activity [48–50]. Therefore, it may be reasonable to conclude that MIA-induced fetal neuroinflammation increases de novo DNMT expression in the prenatal cortex, mediating adaptive DNAm responses to MIA. While DNMT mRNA expression may not always directly correspond to protein expression [83], our demonstration of increased DNMT activity in the GD21 cortex accords with this concept of MIA-induced dysregulation of DNMT-mediated methylation capacity.

In line with these increases in methylation capacity, we observed global hypermethylation in the GD21 cortex of MIA-fetuses. Such DNA hypermethylation-driven gene repression in the fetal brain has been proposed to lead to neurodevelopmental deficits and schizophrenia-like traits [84, 85]. DNAm in the fetal brain is particularly dynamic, with cells undergoing large waves of DNAm changes during cellular development to promote fate-restricted transcriptomes [40–42], leaving these processes particularly vulnerable to environmental-induced disturbances. Collectively, our observations support a hypothesis whereby MIA induces changes in early cellular methylation pathways and DNAm patterns, leading to ongoing alterations in cellular signalling pathways, disturbed neurodevelopment and predisposition to cognitive deficits in MIA-offspring [19]. To our knowledge this is the first evaluation of methylation changes in the fetal brain in response to MIA [21].

### MIA results in changes in DNAm in the adult PFC, but with limited changes in early postnatal development

To gain further insight into how such prenatal changes in methylation pathways may have postnatal effects, we focussed on the PFC. Postnatal age significantly influenced DNMT expression/activity and the pattern of total DNAm. Consistent with the literature [86–88], *Dnmt1* expression was stably expressed across both prenatal and postnatal development while de novo DNMT expression demonstrated distinct temporal changes as development advanced. *Dnmt3b* expression was highest prenatally but displayed the lowest expression of the three *Dnmt* genes postnatally, while *Dnmt3a* expression peaked at PD21, remaining highly expressed throughout postnatal development relative to *Dnmt3b*, patterns consistent with previous findings [86–90]. Likewise, DNMT activity was higher prenatally than postnatally, in agreement with previous studies and the observed developmental expression of *Dnmt* genes [38]. Further, evaluation of global DNAm patterns showed that DNAm increased with age from prenatal to postnatal timepoints, stabilising between adolescence and adulthood [44, 45, 91]. Despite the clear influence of developmental age on DNAm pathways, we found limited MIA-associated changes in these measures postnatally. Indeed, in the PD21 and PD35 PFC, there were no MIA-associated changes in SAM/SAH ratio nor DNMT expression/activity, although there was a trending MIA-induced increase in total DNAm in the male PD21 PFC. However, we did not detect any such effect by PD35. One interpretation of these data is that prenatal MIA-induced changes in methylation pathways are short-lived and not enduring. Alternatively, induced changes during fetal development may exhibit a degree of adaptive plasticity. This could still have neurodevelopmental consequences for cognitive development as normal DNAm is critical during the early postnatal period for synaptic plasticity and learning [92]. In support of the results presented here and from previous studies in MIA-models [21, 62, 93, 94], Basil et al. showed that while increased global DNAm was increased in the hypothalamus, it was unchanged in the striatum of MIA-exposed PD42 mice, yet both regions showing no difference at PD85 [93, 94], showing both regional and age-specific changes in response to MIA.

Surprisingly, we detected changes in methylation pathways in the PD175 PFC, with reduced SAM/SAH ratios, *Dnmt3a/b* mRNA expression and DNMT activity in MIA-offspring. Reduced *Dnmt3a/b* expression together with reduced nuclear DNMT activity in the adult MIA-offspring PFC implies reduced capacity for establishing new DNAm patterns. This may lead to reduced plasticity and memory formation, for which these enzymes are critical [88, 95]. A strength of our study is that we focus on a specific region (PFC), rather than examining whole brains of poly(I:C)-exposed offspring, where no differences in DNMT expression/activity have been observed by a previous study [96], further supporting the regional specificity of epigenetic changes in response to MIA [21, 93, 94]. This approach notably allows us to explore the molecular mechanisms that may contribute to the aetiology of the behavioural changes observed in these MIA-offspring which are governed by the PFC [19]. However, despite observed MIA-induced dysregulation in methylation capacity in the adult PFC, no differences in total DNAm were identified. This, however, does not preclude epigenetic changes at specific gene loci.

### MIA leads to differential methylation of genes involved in neurodevelopment and schizophrenia

A key aim of our study was to investigate the association of epigenetic changes with behavioural dysfunction in response to MIA. Therefore, we examined MIA-induced gene-specific DNAm changes in the adult female PFC, chosen for its essential roles in higher cognitive and executive functions, critical for the ASST in which these females had displayed a deficit [19]. Changes in DNAm have been linked to cognitive performance in schizophrenia [97], and hence evaluation of gene-specific DNAm changes in the adult female PFC may identify pathways important for the observed cognitive phenotype [19]. We did not to analyse the adult male PFC as male offspring did not engage in the ASST [19], which is a limitation given that sex-specific methylomes have been documented both in normal development [98], and schizophrenia [99]. Likewise, we have not analysed gene-specific DNAm changes prenatally as these could not be directly linked to altered behavioural outcomes. The increased global DNAm observed in the fetal cortex raises the interesting question of whether these changes occur at similar genes/pathways to those observed in adulthood.

Despite these limitations, our data support broad MIA-induced genomic DNAm changes in the adult female PFC, with 4030 differentially methylated genes identified, including both hypermethylated and hypomethylated genes. This demonstrates that the prenatal insult of MIA can have enduring epigenetic consequences. A study of neuronal DNAm changes in schizophrenia suggested that schizophrenia-associated differential methylation accounted for up to 40% of schizophrenia-associated differentially expressed genes [91], suggesting that the observed MIA-induced DNAm changes here may also drive alterations in the cortical transcriptome and cortical function.

Our analyses showed that differentially methylated genes were functionally involved in neurodevelopmental processes, including synapse and axon generation, neuronal signalling and cellular differentiation, and in schizophrenia-associated behaviours, including anxiety, learning, memory and cognition. Differentially methylated genes also showed a significant enrichment for schizophrenia risk genes alongside a significant overlap with those identified by Richetto and colleagues, who analysed MIA-induced DNAm changes in the adult male PFC in a mouse poly(I:C)-model [62]. In line with our study, re-analysis of the Richetto dataset also demonstrated a significant enrichment for schizophrenia risk genes [100], supporting the relevance of the findings from both these models for this disease phenotype. The overlap between these studies also suggests that the genes identified here exhibit robust methylation changes in response to MIA across sexes and species and are therefore highly likely to be functionally relevant in the pathogenesis of the adult behavioural phenotype [19] and are thus likely important mediating pathways linking MIA to schizophrenia phenotypes.

Although neuro-molecular alterations in response to MIA are undoubtedly complex, involving numerous transcriptomic and epigenetic changes ([19–21], and this study), identification of key pathways which are robustly affected may confer mechanistic insights regarding individual susceptibility to schizophrenia. Therefore, exploration of the MIA-affected pathways identified here and how they contribute to schizophrenia-like behavioural traits across development will hence be a focus of future work and may offer promise as future therapeutic targets.

## Supplementary information

Supplementary Information Summary

Supplement S1: Supplementary Methods and Results

Supplement S2: Maternal Immune Activation (MIA) Model Reporting Guidelines Checklist.

Supplement S3: Chromosomal location of RRBS-identified DMCs and DMRs.

Supplement S4: CpG context annotation of filtered DMCs and DMRs.

Supplement S5: Gene annotation of filtered DMCs and DMRs.

Supplement S6: Gene ontology and KEGG pathway analysis.

## Data Availability

All sequencing output data sets generated in this study are included in the Supplemental Information of this article. All other data sets are available from the corresponding author on reasonable request.
