# Supplementary material for: Developmental modulation of schizophrenia risk gene methylation in offspring exhibiting cognitive deficits following maternal immune activation
Source: Mol Psychiatry. 2025 Aug 29;31(1):418–29. doi: 10.1038/s41380-025-03147-1 (PMC12700827; doi:10.1038/s41380-025-03147-1)
Supplement: Supplementary file 1 — Supplementary Information Summary [file 41380_2025_3147_MOESM1_ESM.pdf]

Supplementary Information

Supplement S1: Supplementary methods and results. This supplement contains additional information on methods and results presented in the main text.

Supplement S2: Maternal Immune Activation (MIA) Model Reporting Guidelines Checklist. This supplement contains the completed checklist relating to the methodological details of the animal model used in this study.

Supplement S3: Chromosomal location of RRBS-identified DMCs and DMRs. This supplement contains four Supplementary Tables describing the chromosomal positions of unfiltered and filtered DMCs (Table S3.1 and S3.2, respectively) and DMRs (Table S3.3 and S3.4, respectively).

Supplement S4: CpG context annotation of filtered DMCs and DMRs. This supplement contains two Supplementary Tables denoting the CpG context annotation of filtered DMCs (Table S4.1) and DMRs (Table S4.2).

Supplement S5: Gene annotation of filtered DMCs and DMRs. This supplement contains four Supplementary Tables denoting gene (promoter, exon and intron) and intergenic annotations of filtered DMCs (Table S5.1 and S5.2, respectively) and DMRs (Table S5.3 and S5.4, respectively).

Supplement S6: Gene ontology and KEGG pathway analysis. This supplement contains four Supplementary Tables denoting significant mapping of differentially methylated genes to gene ontologies of biological processes (Table S6.1), molecular functions (Tables S6.2) and cell component (Table S6.3) and KEGG pathways (Table S6.4).
