# Supplementary material for: Developmental modulation of schizophrenia risk gene methylation in offspring exhibiting cognitive deficits following maternal immune activation
Source: Mol Psychiatry. 2025 Aug 29;31(1):418–29. doi: 10.1038/s41380-025-03147-1 (PMC12700827; doi:10.1038/s41380-025-03147-1)
Supplement: Supplementary file 2 — Supplement S1: Supplementary Methods and Results [file 41380_2025_3147_MOESM2_ESM.pdf]

Supplement S1. Supplementary methods and results

S1. Supplementary methods

S1.1. Animal procedures

All animals used in this study were from our previous published cohorts (17,19). All animal experiments and procedures were conducted under the authority of project licence P473EC3B1, in accordance with the Animals in Scientific Procedures Act (ASPA) 1986 and locally approved by the Animal Welfare and Ethical Review Body (AWERB) at the University of Manchester. Complete details of the animal study have also been reported using the Maternal Immune Activation (MIA) Model Reporting Guidelines Checklist devised by Kentner et al. (51) as an adaptation of the ARRIVE guidelines in Supplement S2. Figure S1.1 shows a timeline of the animal study.

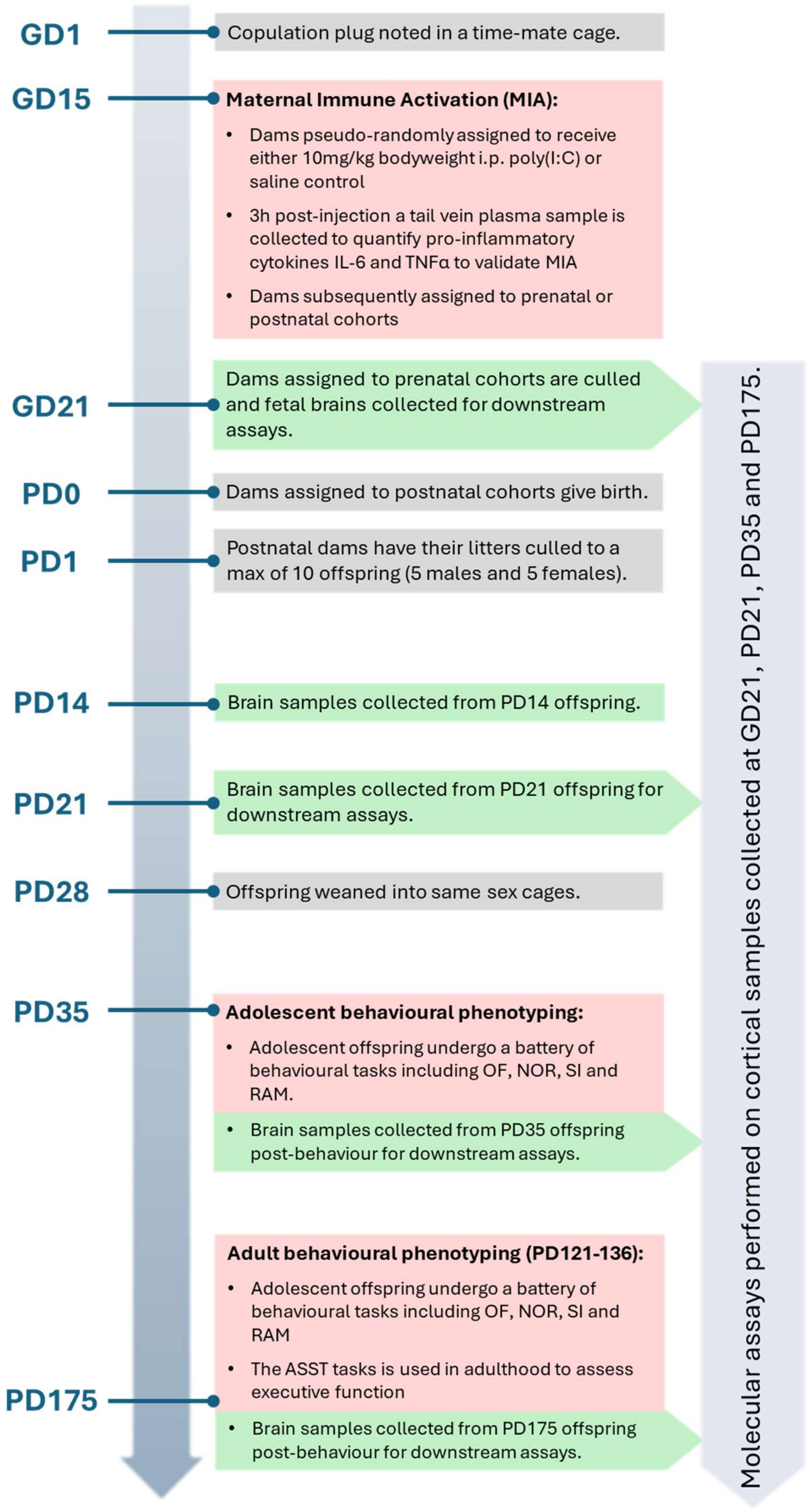

Figure S1.1. Study Timeline
Timeline depicting the developmental stages and outcome measures of the study. Left shows the various gestational days (GD) and postnatal days (PD) timepoints of fetal/offspring life. Grey boxes indicate husbandry procedures. Red boxes indicate experimental procedures including induction of MIA on GD15 and offspring behavioural assessment at PD35 and PD175. Green boxes indicate collection of brain samples and those arrowed point to subsequent molecular assay processing.

a) Animal Husbandry

Adult Wistar rats were obtained from Charles River Laboratories, UK, and maintained in the Biological Services Facility at the University of Manchester within two-level individually ventilated cages (Double-Decker Cage; Tecniplast, Italy), with up to five animals per cage. Throughout experiments, animals were maintained on a 12h light:dark cycle (07:00-19:00) at a temperature of 21 ± 2°C and humidity 55 ± 5% with *ad libitum* access to standard rat chow (Special Diet Services, Essex, UK) and water.

b) Maternal immune activation (MIA)

Nulliparous female rats (260.6 ± 2.70g; mean ±SEM) were time-mated with one randomly assigned male for up to three days, and mating cages were checked regularly for presence of a copulation plug. Evidence of a copulation plug was taken as confirmation of conception and termed gestational day (GD) 1. Following successful mating, pregnant females were pair-housed until GD15 when dams were pseudo-randomly assigned to receive 10mg/kg bodyweight endotoxin-free, low molecular weight (LMW) poly(I:C); tlrc-picw; InvivoGen, London, UK), reconstituted in physiological saline (0.9% NaCl) or saline only (vehicle control). Experimenters involved in treatment administration and data collection were blinded to treatment group until all data had been collected. Poly(I:C) was reconstituted by an independent experimenter who did not otherwise take part in data collection. A total of 30 dams received injections (N=15 vehicle and N=15 poly(I:C)). Solutions were delivered via a single intraperitoneal (i.p.) injection performed between 08:00-10:00 to minimise circadian influence on immune outcomes (52). All treated dams were randomly assigned by random number generation to either prenatal (N=12) or postnatal cohorts (N=18). It was predetermined that any dams with significantly small litters (<2 standard deviations from the mean) would be excluded from analysis due to abnormal maternal nutrient resource provision (19).

c) Quantification of maternal plasma cytokines

3h post-injection plasma was collected from lateral tail vein blood samples for analysis of downstream inflammatory markers using IL-6 (ab100772) and TNFα (ab100784) ELISA kits (both Abcam, Cambridge, UK) following the manufacturer’s instructions. Confirming our previous results (17,19), dams exposed to poly(I:C) showed a significant elevation in maternal pro-inflammatory cytokines IL-6 (GLM: $F_{1,25}$=4.69, p=0.040; Figure S1.2A) and TNFα (GLM: $F_{1,23}$=37.82, p<0.001; Figure S1.2B) at 3h post-injection, indicative of an acute MIA response.

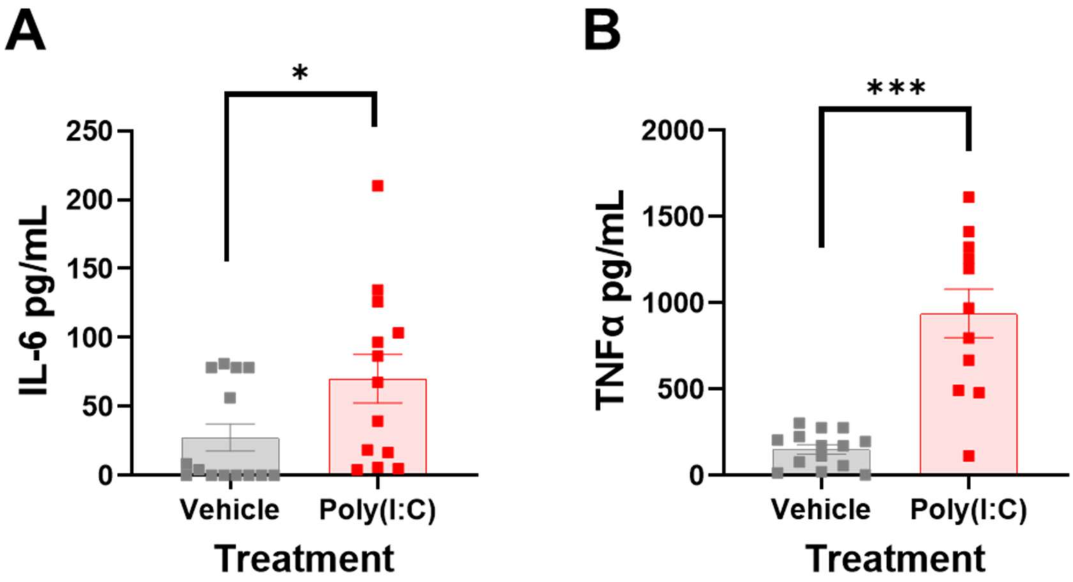

Figure S1.2. Poly(I:C) elicits increased maternal plasma cytokine concentrations at 3h post-treatment.
A. IL-6 (N=27: N=14 Vehicle and N=13 poly(I:C)). **B.** TNFα (N=25 N=14 Vehicle and N=11 poly(I:C)). Samples were analysed from dams which met the criteria for inclusion in the study and for which there was sufficient plasma volume to perform the ELISA. Data was analysed using a general linear model with significant main effects of treatment *p<0.05, ***p<0.001.

d) Sample collection

Table S1.1 shows a distribution of each dam and litter and their experimental assignment for the purposes of this study.

Dams assigned to prenatal cohorts were anaesthetised with 5L/min isoflurane (Abbott, Maidenhead, UK) in 2L/min O_2 until cessation of breathing and then sacrificed by cardiac puncture and removal of the heart. Their abdominal cavity was opened, the uterine horn exposed, and the fetoplacental unit rapidly removed. Fetal tail tips were taken for sex-typing using the KAPA Express Extract and KAPA2G Robust HotStart® ReadyMix™ (Sigma-Aldrich, Gillingham, UK) as described previously, using the male-specific *Sry* gene to identify male sex (17). Fetal whole brains were then rapidly harvested and weighed and processed for downstream molecular analysis.

Dams assigned to postnatal cohorts were allowed to birth naturally with presence of pups in the nest considered postnatal day (PD) 0. On PD1, all pups were removed from the nest and sexed based on ano-genital distance (53). To ensure even distribution of maternal care resources across litters (54), litters were culled to 10 with equal sex ratios maintained where possible. The remaining pups were returned to the nest with the dam and assigned to be culled on PD14, PD21, PD35 and PD175. These animals were asphyxiated by 2L/min CO_2, followed by cervical dislocation with brains also isolated for downstream molecular analysis. An individual's assignment to either culling at PD1, 14, 21, 35 or 175, or to behavioural assessment was randomly decided by random number generation with at least one male and one female from each litter assigned to a single timepoint where possible to minimise litter effects. Note that the PD1 and PD14 samples were not included in this study (Figure S1.1). Experimenters were blinded to treatment group throughout sample processing and molecular testing.

e) Behavioural testing

Behavioural testing was performed postweaning (weaning at PD28) for adolescent (PD35) and adult (PD121-134) offspring as described in Potter et al. (19). Experimenters were blinded to offspring treatment group during behavioural testing and scoring. Behavioural tasks were novel object recognition (NOR) and elevated plus maze (EPM) at PD35, followed by NOR, EPM, social interaction (SI), the attentional set-shifting task (ASST), and the radial arm maze (RAM) in adulthood (PD121-134). Key results showed that poly(I:C) exposure induced deficits in adult female socialisation (SI pro-social sniffing and following) and cognitive flexibility (ASST). Furthermore, the maternal TNFα response

significantly predicted offspring socialisation (SI), cognitive flexibility (ASST), and spatial working memory (RAM) deficits. It should be noted that adult (PD121-134) male offspring did not successfully engage in the ASST. Hence, we were unable to determine any cognitive deficit in male offsrping. Adolescent offspring were culled immediately post-behaviour on PD35 while adults were standardised to be culled on PD175.

Table S1.1. Summary of dams and litter sizes

Cohort assignment	Treatment group	Litter size as number of fetuses/pups	Sex distribution as number per litter	Assignment to cull**	Behavioural assessments***	Brain samples used in molecular analyses for this study****
Prenatal<br>(N=12 dams)	N=6 VEH*	Mean ±SD: 13±3<br>Range:3-19	**Males:**<br>Mean ±SD: 6±2<br>Range 1-10	GD21: Whole litter collected<br>*Note: at this stage small litters were excluded from any further analysis**	NA	n=7 males (1-2/litter)
			**Females:**<br>Mean ±SD: 7±2<br>Range 2-9			n=5 females (1/litter)
	N=6 PIC	Mean ±SD: 13±2<br>Range:10-16	**Males:**<br>Mean ±SD: 6±2<br>Range 3-9			n=6 males (1/litter)
			**Females:**<br>Mean ±SD: 6±1<br>Range 4-9			n=6 females (1/litter)
Postnatal<br>(N=18 dams)	N=9 VEH	**At birth**:<br>Mean ±SD: 13±2<br>Range:10-16<br><br>**After PD1 culls:**<br>Mean ±SD: 10±0<br>Range: all litters=10	**Males at birth:**<br>Mean ±SD: 7±2<br>Range 4-8<br><br>**Males after PD1 cull:**<br>Mean ±SD: 5±1<br>Range: 4-8	PD1: n=7 (up to 3/litter)<br>PD14: n=10 (up to 2/litter)<br>PD21: n=11 (up to 2/litter)<br>PD35: n=13 (up to 2/litter)<br>PD175: n=14(up to 3/litter)	PD35: n=21<br>(up to 3/litter)<br><br>PD175: n=9<br>(up to 2/litter)	PD21: n=6 (up to 1/litter)<br>PD35: n=7 (up to 1/litter)<br>PD175: n=7 (up to 1/litter)
			**Females at birth:**<br>Mean ±SD: 5±2<br>Range: 2-12<br><br>**Females after PD1 cull:**<br>Mean ±SD: 5±1<br>Range: 2-6	PD1: n=25 (up to 6/litter)<br>PD14: n=8 (up to 1/litter)<br>PD21: n=11 (up to 2/litter)<br>PD35: n=13 (up to 2/litter)<br>PD175: n=14 (up to 2/litter)	PD35: n=27<br>(up to 4/litter)<br><br>PD175: n=14<br>(up to 3/litter)	PD21: n=6 (up to 1/litter)<br>PD35: n=7 (up to 1/litter)<br>PD175: n=7 (up to 1/litter)

	N=9 PIC*	**At birth:**<br>Mean ±SD: 12±3<br>Range:5-16<br><br>**After PD1 culls:**<br>Mean ±SD: 9±2<br>Range:8-10<br>*Note: 7 litters were n=10, with one litter of n=8 after litter exclusion (excluded litters were culled at PD1 and excluded from any further analysis)*.*	**Males at birth:**<br>Mean ±SD: 6±2<br>Range 3-10<br><br>**Males after PD1 cull:**<br>Mean ±SD: 5±1<br>Range: 5-7	PD1: n=15 (up to 4/litter)<br>PD14: n=7 (up to 1/litter)<br>PD21: n=13 (up to 2/litter)<br>PD35: n=13 (up to 2/litter)<br>PD175: n=11 (up to 2/litter)	PD35: n=25<br>(up to 4/litter)<br><br>PD175: n=11<br>(up to 2/litter)	PD21: n=7 (up to 1/litter)<br>PD35: n=7 (up to 1/litter)<br>PD175: n=6 (up to 1/litter)
			**Females at birth:**<br>Mean ±SD: 8±3<br>Range 2-7<br><br>**Females after PD1 cull:**<br>Mean ±SD: 4±1<br>Range: 3-5	PD1: n=7 (up to 2/litter)<br>PD14: n=7 (up to 1/litter)<br>PD21: n=10 (up to 2/litter)<br>PD35: n=9 (up to 2/litter)<br>PD175: n=10 (up to 2/litter)	PD35: n=20<br>(up to 3/litter)<br><br>PD175: n=10<br>(up to 2/litter)	PD21: n=6 (up to 1/litter)<br>PD35: n=6 (up to 1/litter)<br>PD175: n=6 (up to 1/litter)

Data in the table are represented throughout as mean ±standard deviation (SD) for number of dams (N) or fetuses/offspring (n) in each treatment group, litter and sex distribution including assignment to downstream assay.

*One prenatal vehicle dam and one postnatal poly(I:C) dam produced significantly small litters (N=3 and N=5, respectively) compared to the cohort average and were excluded from behavioural and molecular analysis following our predefined criteria resulting in N=11 dams in the prenatal cohort and N=17 dams in the postnatal cohort included in the presented analyses.

**Offspring culled at PD1 and PD14 were not included in any analyses in this study.

***Details of behavioural tasks conducted and outcomes are described in our previous paper (19).

****Samples selected for use in this study were randomly determined, with primarily one male and female selected from each litter at each timepoint to achieve the required n-number (see Section S1.5 for sample size calculations).

1.2. Preparation of isolated brains for downstream analysis

For nucleic acid and protein analysis, whole brains collected at GD21, PD21, PD35 and PD175 were bisected into left and right hemispheres and stored in RNAlater (Sigma-Aldrich, Gillingham, UK) for nucleic acid isolation or flash-frozen using dry ice for protein isolation. Hemispheric dissections were subsequently performed on ice to isolate brain regions of interest as directed by the Rat Brain Stereotaxic Co-ordinates (55). For GD21, when the PFC has not yet formed (56), the left or right hemisphere frontal cortex (FC) only were dissected (bregma 5.64-3.00, interaural 14.64-12.00), whereas from later postnatal samples (PD21, PD35, PD175), the PFC was dissected (bregma 5.16-2.52; interaural 14.16-11.52). All dissected brain tissues were stored in RNAlater (left hemisphere for nucleic acid isolation) or frozen without buffer (right hemisphere for protein isolation). The PFC was selected for its involvement in cognition and schizophrenia pathophysiology and, particularly, its role in the ASST (57,58) as we have previously shown that offspring of poly(I:C)-treated dams exhibit a cognitive deficit in adulthood (19).

S1.3. Real-time quantitative PCR (qPCR)

RNA (850ng input) was reverse transcribed to complementary DNA (cDNA) using the QuantiTect Reverse Transcription Kit (Qiagen, Manchester, UK) according to manufacturer's instructions. Relative gene expression was quantified by qPCR using pre-optimised Qiagen QuantiTect Primers (Table S1.2), and QuantiFast SYBR Green RT-PCR Kit (Qiagen, Manchester, UK) following the manufacturer's instructions. All qPCR experiments were performed on the AriaMx System (Agilent, Cheadle, UK).

To normalise candidate gene expression, the stability of expression for six common reference genes *Gapdh, Ubc, Ywhaz, B2m, Actb, Mdh1* (GeNorm primer panel, Z-HK-SY-RA-600; PrimerDesign Ltd., Chandler's Ford, UK) were analysed using a random selection of samples across treatment group, sexes and age. Raw Ct values were uploaded into Qbase+ GeNorm analysis software (Biogazelle, Ghent, Belgium) and GeNorm analysis was performed as described by Vandesompele et al. (59). Based on this analysis, the three most stable reference genes (*Gapdh, Ubc* and *Mdh1*) were used for expression normalisation. Normalised candidate gene expression was calculated by dividing the sample candidate gene expression by the geometric mean of the reference gene expression values.

Table S1.2. QuantiTect primer assays for candidate genes

Gene	QuantiTect primer assay (Cat no.)	Amplicon size (bp)	Exons spanned
Dnmt1	Rn_RGD:620979_1_SG (QT00493577)	81	10/11/12
Dnmt3a	Rn_Dnmt3a_1_SG (QT01567083)	61	11/12
Dnmt3b	Rn_Dnmt3b_1_SG (QT01584625)	144	3/4

Abbreviation: bp, base pair

S1.4. Reduced representation bisulphite sequencing (RRBS) and bioinformatics

a) Library preparation

The PFC of adult females was used in RRBS analysis to identify gene-specific changes in DNAm. Adult females were chosen as we have previously demonstrated (19; Section S1.1e) that poly(I:C) exposure resulted in a deficit in the ASST in these offspring, reminiscent of the executive function deficits observed in schizophrenia patients.

RRBS library preparation from PFC DNA samples (four vehicle and four poly(I:C) female offspring), sequencing and standard bioinformatics was performed by Diagenode (Liège, Belgium). DNA concentration was measured using the Qubit® dsDNA BR Assay Kit (ThermoFisher, Waltham, USA). DNA quality was assessed with the Fragment Analyzer™ and the DNF-488 High Sensitivity genomic DNA Analysis Kit (Agilent, Cheadle, UK). All eight adult female PFC DNA samples passed Diagenode quality control and were used for library preparation. 100ng PFC DNA was used for library preparation, with spike-in controls (methylated and non-methylated) used to assess sequencing efficiency. RRBS libraries were prepared using the Premium Reduced Representation Bisulphite Sequencing Kit (Diagenode, Liège, Belgium) following the manufacturer's instructions and published workflow (61). The final prepared amplified library pool concentration was measured using the Qubit® dsDNA HS Assay Kit (Thermo Fisher Scientific, Waltham, USA) and the fragment profile checked using the DNF-474 NGS fragment kit and Fragment Analyzer (Agilent). Fragments 100-1000bp, with an average fragment size of 333 bp were obtained (Figure S1.3). The RRBS library pool was then sequenced in paired-end mode on an Illumina NovaSeq 3000/4000 (Illumina, San Diego, USA), generating 50 base reads (SE50).

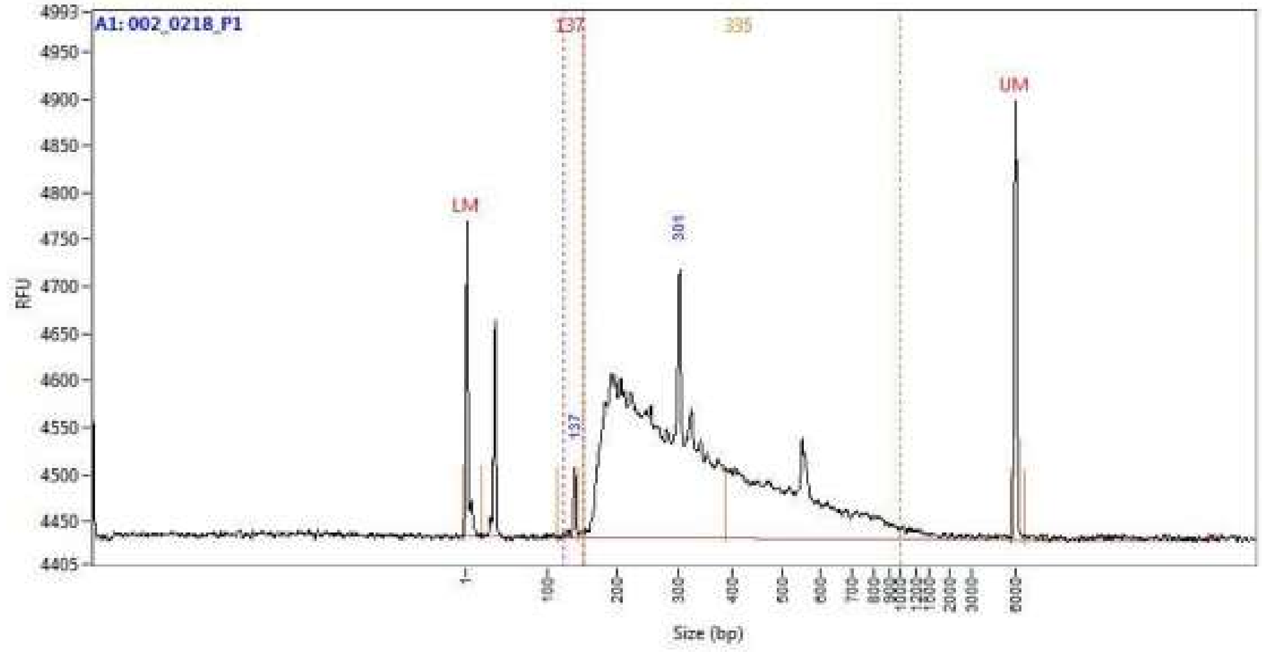

Figure S1.3. Electropherogram of RRBS library pool
Electropherogram showing various fragments from 100-1000bp for sequencing, with an average fragment size of 335bp.

b) Bioinformatics

Quality control of sequencing reads was performed using FastQC v0.11.8 (Andrews, 2010) and adapter removal was performed using Trim Galore! v0.4.1. (64). Sequence reads were aligned to the rat reference genome Rn5.0 using Bismark v0.20.0 (65). Mapping efficiency was calculated by the number of uniquely mapped sequence reads divided by the total number of reads, with a mapping efficiency 55-70% expected due to sequence redundancy following bisulphite conversion. The cytosine2coverage and bismark_methylation_extractor modules of Bismark were used to infer methylation state (percentage methylation) of all uniquely mapped cytosines. The reported cytosines were subsequently sorted to isolate only CpGs captured in all eight samples. The spike-in control sequences (methylated and non-methylated) were used to assess the bisulphite conversion rates to validate the efficiency of the bisulphite treatment, with ≤2% and ≥98% conversion rates considered acceptable, respectively.

Methylkit v1.7.0 an R/Bioconductor package, was used for differential methylation analysis between the two sample groups (Poly(I:C) vs Vehicle). The dataset was first sifted to discard low coverage CpGs (with coverage <10X in all samples as these reads are less accurate) and for notably high coverage CpGs (CpGs with coverage > 99.9th percentile were discarded as they are likely indicative of a PCR bias to these sequences). A principal component analysis (PCA) followed by hierarchical clustering was then performed to interpret sample similarity. Following initial CpG sifting and similarity testing, a pairwise comparison was performed for 'Poly(I:C)' versus 'Vehicle' to identify differentially methylated individual CpGs (DMCs) and differentially methylated regions (DMRs), the latter comprising a sequence stretch of 1000bp. Logistic regression was used to compare statistical significance of methylation differences between groups at each given DMC/DMR. After p-values were computed, the sliding window model (SLIM) was used to correct p-values to q-values, accounting for multiple comparison tests. Statistically significant DMCs and DMRs were identified with a pre-determined q-value cut-off ≤0.01 and a methylation difference ≥25%. These stringent cut-offs were used to account for false discovery of DMCs due to technical variability (e.g., coverage, read depth) and inherent heteroscedasticity of raw methylation values (61,66,67). Filtered and unfiltered DMCs and DMRs are shown in Supplement S3. All DMCs and DMRs were annotated to genomic location with the R/Bioconductor package annotatr (68), with the refGene and CpG island annotations from UCSC Rat reference genome Rn5.0 (69). The annotation comprised two categories: (i) distance to a CpG island including: overlapping a known CpG island; within the 2000bp flanking region of a CpG island (CpG

shore); within 2000bp of the CpG shore (CpG shelves) or outside these regions (Open sea) and (ii) genic annotation (intergenic, exonic, intronic or promoter).

Using the genic location, DMCs and DMRs were mapped to genes in which promoter or gene body they were located. Gene ontology was performed to determine the enriched biological processes, molecular functions and cellular components of the genes associated with DMCs/DMRs using the R/Bioconductor topGO package (70). Reactome Pathway enrichment analysis was also carried out with the R/Bioconductor package ReactomePA (71) which uses KEGG (Kyoto Encyclopedia of Genes and Genomes) terms for the analysis. Enrichment analyses was performed using hypergeometric model with false discovery rate used to correct p-values.

Finally, to assess the validity of the RRBS gene list for schizophrenia research, schizophrenia-risk gene lists were collated from published genetic and epigenetic studies (Table S1.3). Gene identifiers across the compiled gene lists were converted to rat orthologues using the g:Profiler database (75). A Fisher's exact test was used to assess enrichment of the RRBS gene list against each of the compiled gene lists in Table S1.3.

Table S1.3. Gene lists associated with schizophrenia

Gene List ID	Summary of Gene List	Data Reference(s)*
**SZ_GENE**	Genes with a genetic link to schizophrenia from GWAS, WES, CNV and linkage studies	72,73
**SZ_EXP**	Genes differentially expressed in schizophrenia	72,74
**SZ_METH**	Genes differentially methylated in schizophrenia	29,72
**MIA_WGBS**	Differentially methylated CpG sites and regions identified in the adult male PFC following poly(I:C)-exposure in mice on GD9 or GD17	62

*Comparison gene lists were extracted from the schizophrenia database SZDB v2.0 (72) and updated for studies published since the database update in 2020.

S1.5. Statistical analysis of molecular data

a) Sample size calculation

Sample size was calculated using G*Power (v3.1.9.2. Düsseldorf, Germany), employing a sensitivity analysis. We estimated a medium-large effect size (f=0.25-0.4) for molecular changes based on previous work from our and other molecular studies in MIA models (17-20,62). This analysis showed a required minimum of 5-6 offspring/sex/group/age was for molecular analysis to be sufficiently powered ($1-\beta=0.8$), with a type I error rate (α)=0.05. Dam numbers are based on required sample sizes of offspring from these power calculations.

b) Statistical analysis of molecular data (excluding RRBS)

Statistical analysis of fetal/offspring molecular outputs (excluding RRBS) were performed using SPSS v28.0 (IBM). Within-group outliers (i.e. within each sex and group) for all molecular measures were identified and excluded from statistical analysis using the built-in SPSS extreme outlier function.

When analysing molecular changes in response to MIA, between-group analysis of molecular outputs used a general linear mixed model (GLMM), including dam as a random factor and the following predictors: fixed factors (sex, group) and co-variates (maternal IL-6 and TNFα), with p-values≤0.05 considered statistically significant and $0.05<p\leq0.075$ highlighted as approaching significance. For all GLMMs, where sex or group*sex interactions were significant or approaching significance, a post-hoc GLMM was performed within a single sex with group as a fixed factor and maternal cytokines as co-variates. Where appropriate, correlations were used to evaluate the relationship between quantitative measures. For correlations, normality of distribution was determined using the Shapiro-Wilk Test. When both variables were normally distributed, post-hoc Pearson's correlations were used; where one or more variables were non-normally distributed, Spearman's (rho) correlations were used.

For all GLMM analyses the degrees of freedom were estimated using the Satterthwaite approximation and with normality of error assumptions assessed. Residuals of full models were examined for obvious deviations from normality and for heteroscedasticity and homogeneity of variance issues using residual against fitted value plots. Within-group and between-group variation is shown in figures using SEM.

c) Generation of figures

For bar charts, data are presented as mean ± SEM (GraphPad Prism (v9.0)), with numbers of dams per group (N) and fetuses/offspring per sex per group (n) indicated in the Figure legends. Sex*group interactions are not indicated on Figures, but rather the post-hoc within-sex analyses are shown.

S2. Supplementary Results

S2.1. RRBS quality control checks

The initial RRBS sequencing quality control checks demonstrated that the sequencing reads are within the expected ranges: mapping efficiency of 65.7-67.09%, with ≥75% of detected CpGs covered more than 10X such that they could be included for differential analysis, resulting in 721,189 detected CpGs covered ≥10X common to all eight samples (Table S1.4). Spike-in controls all passed within acceptable limits: ≤1.93% (methylated) and ≥99.31% (non-methylated) conversion rates (Table S1.4). For each sample the CpG read depth and distribution of percentage methylation per CpG sites can be found in Figures S1.4 and S1.5, respectively.

Table S1.4. Sequencing sample overview

Sample ID	Total Read Pairs	Uniquely aligned Reads	Mapping efficiency (%)	CpGs detected	CpG Covered >10	Average Coverage	Conversion rate of methylated spike (%)	Conversion rate of non-methylated spike (%)
**FVEH1 (FA1)**	27,930,930	18,646,038	66.76	2,182,574	1,645,383	30.06X	1.71	99.96
**FVEH2 (FA2)**	35,757,041	23,494,127	65.70	2,439,341	1,880,602	34.09X	1.53	99.83
**FVEH3 (FA3)**	34,382,801	22,883,318	66.55	2,428,545	1,842,391	31.72X	1.72	99.77
**FVEH4 (FA4)**	37,528,364	24,718,122	65.87	2,415,256	1,876,946	36.70X	1.71	99.74
**FPIC1 (FB1)**	42,555,265	28,047,116	65.91	2,482,566	1,973,325	40.86X	1.71	99.31
**FPIC2 (FB2)**	39,047,620	26,106,922	66.86	2,440,463	1,926,744	38.34X	1.87	99.75
**FPIC3 (FB3)**	37,643,426	25,254,916	67.09	2,346,579	1,888,549	39.06X	1.93	99.83
**FPIC4 (FB4)**	33,718,054	22,356,976	66.31	2,394,051	1,831,638	32.05X	1.77	99.62

Abbreviations: F, female; VEH, vehicle; PIC, poly(I:C).

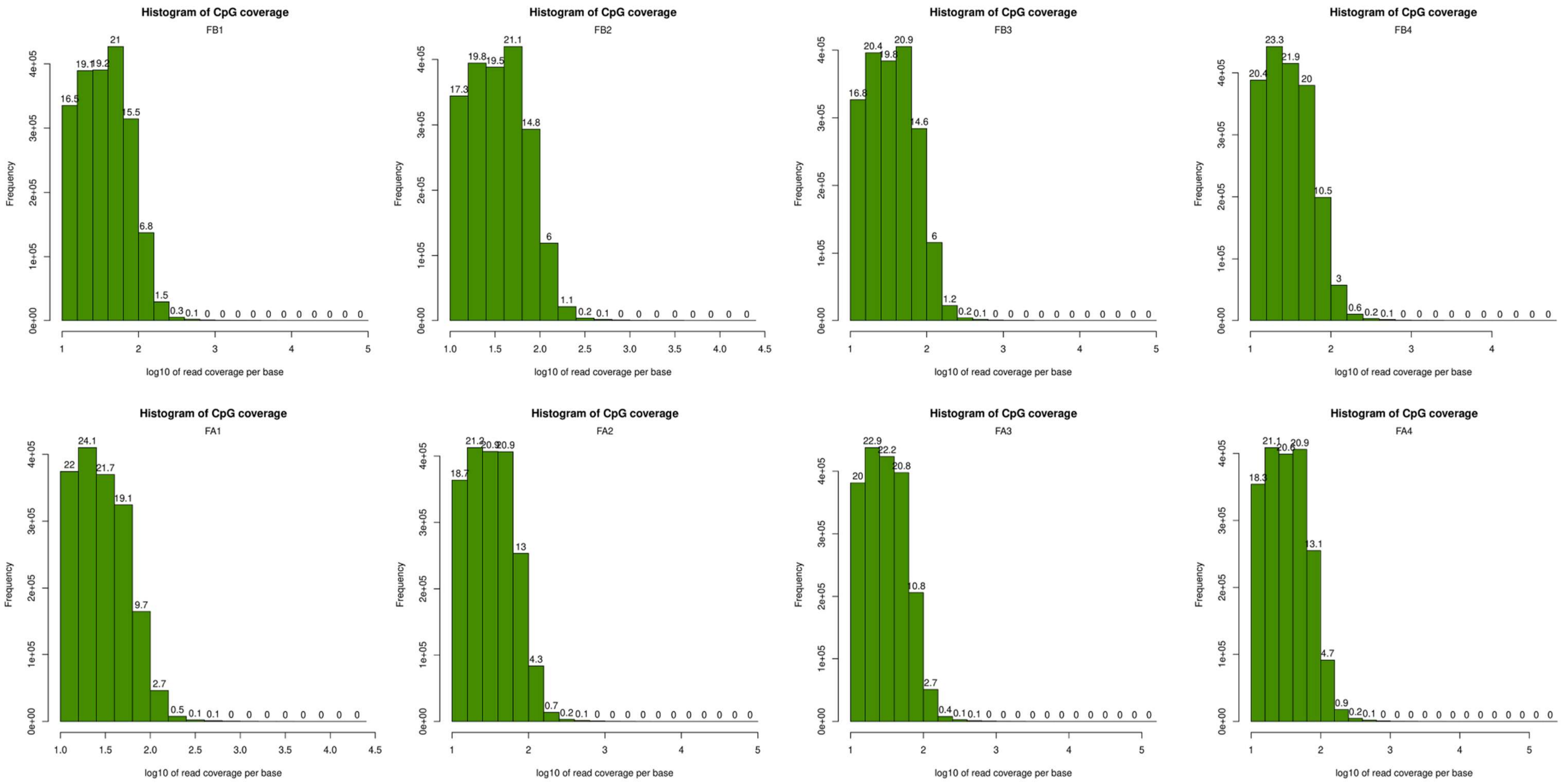

Figure S1.4. Histograms detailing read coverage of each CpG detected within each sample.
FB samples indicate poly(I:C) samples (n=4), FA samples indicate vehicle samples (n=4). The read depth/coverage are binned and displayed on the x-axis (log10 scale) and indicate the total number of bases sequenced and mapped at a given reference base position, while the total numbers of reference bases that occupy each read depth bin are displayed on the y-axis. Numbers on bars denote what percentage of reference locations are contained in that bin.

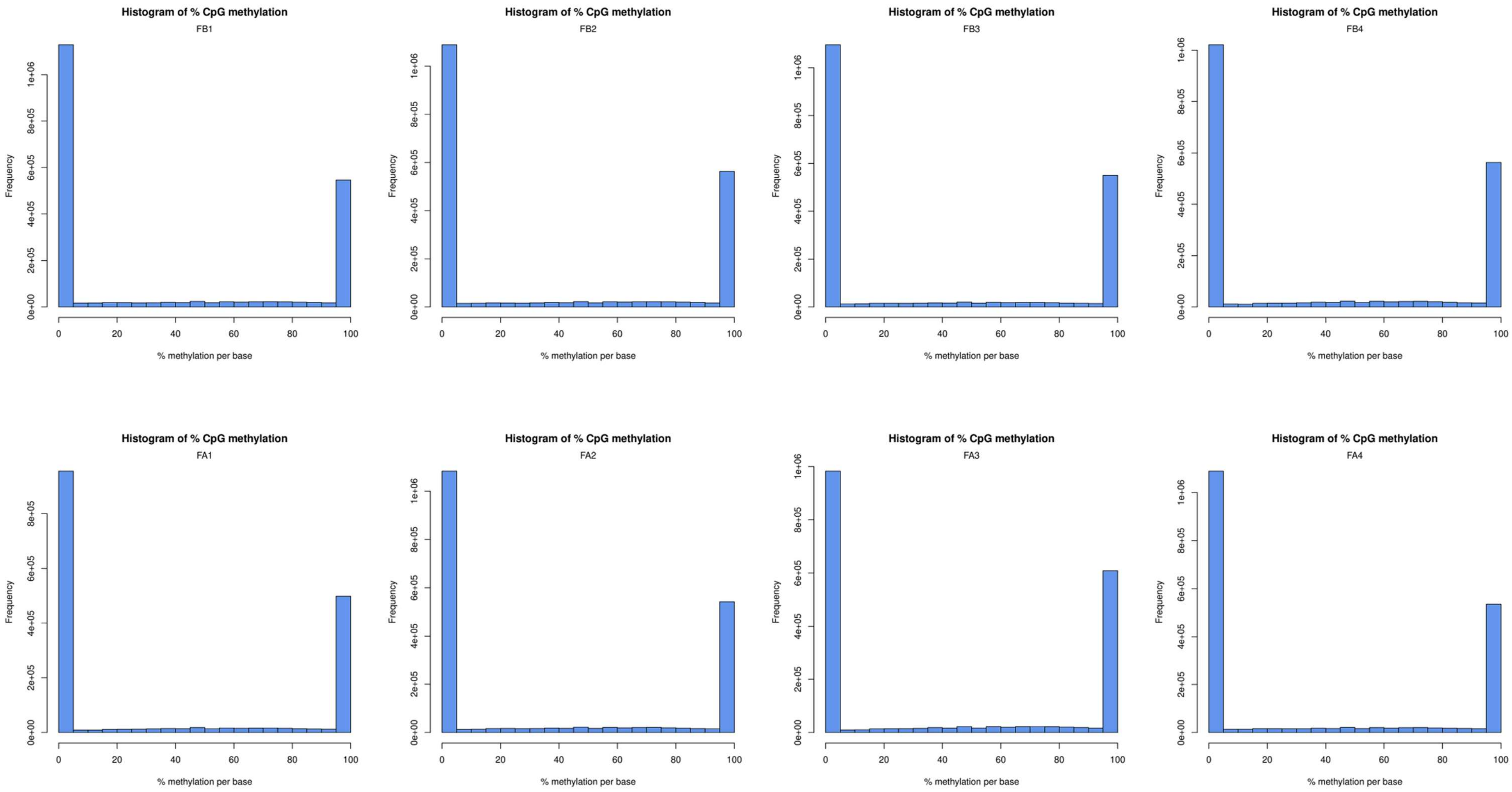

Figure S1.5. Histograms detailing the percentage (%) methylation of each CpG detected across within each sample.
FB samples indicate poly(I:C) samples (n=4), FA samples indicate vehicle samples (n=4). Percentage methylation of bases are binned and displayed on the x-axis and indicate the total number of CpG sites sequenced with that methylation percentage, while the total numbers of CpG sites that occupy each methylation bin are displayed on the y-axis.

S2.2. Differential methylation analysis

Using the methylation profile of each sample (based on the computed methylation percentage at each CpG) was used be to explore sample similarity PCA (Figure S1.6) followed by hierarchical clustering (Figure S1.7). Subsequently a pairwise comparison was used to identify DMCs and DMRs between the two sample groups and establish whether these were hypomethylated or hypermethylated (Poly(I:C) vs Vehicle; Figure S1.8). Next, logistic regression was used to determine the statistical significance percentage difference in methylation and significance between the two groups. Table S3.1 and Table S3.2 (Supplement S3) depict all identified DMCs and DMRs between poly(I:C) relative to vehicle groups. These DMCs/DMRs were then filtered using the pre-determined cut-offs (q-value≤0.01; methylation difference ≥25%), resulting in 22,096 DMCs and 3,227 DMRs used for downstream analysis (Table S3.3 and S3.4, respectively; Supplement S3). Of the DMCs 12,985 (58.8%) were hypomethylated and 9,111 (41.2%) were hypermethylated, while for DMRs 2,025 (62.8%) were hypomethylated and 1,202 (37.2%) were hypermethylated.

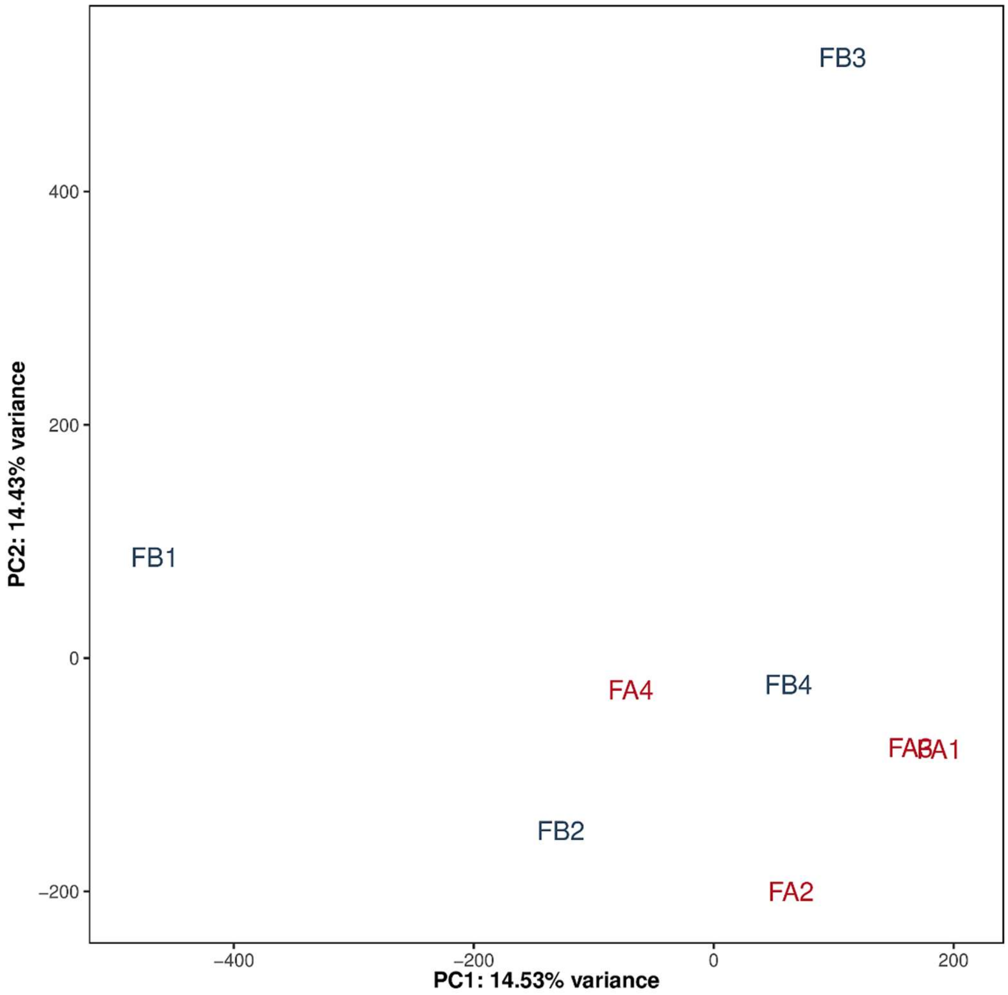

Figure S1.6. Principal component analysis (PCA) of RRBS samples.
FB samples indicate poly(I:C) samples (n=4; red), FA samples indicate vehicle samples (n=4; grey). The PCA shows the methylation profiles of the study samples. Each sample is represented by a its name, the axes are the first two PCs, the percentages indicate the fraction of variance explained by each PC.

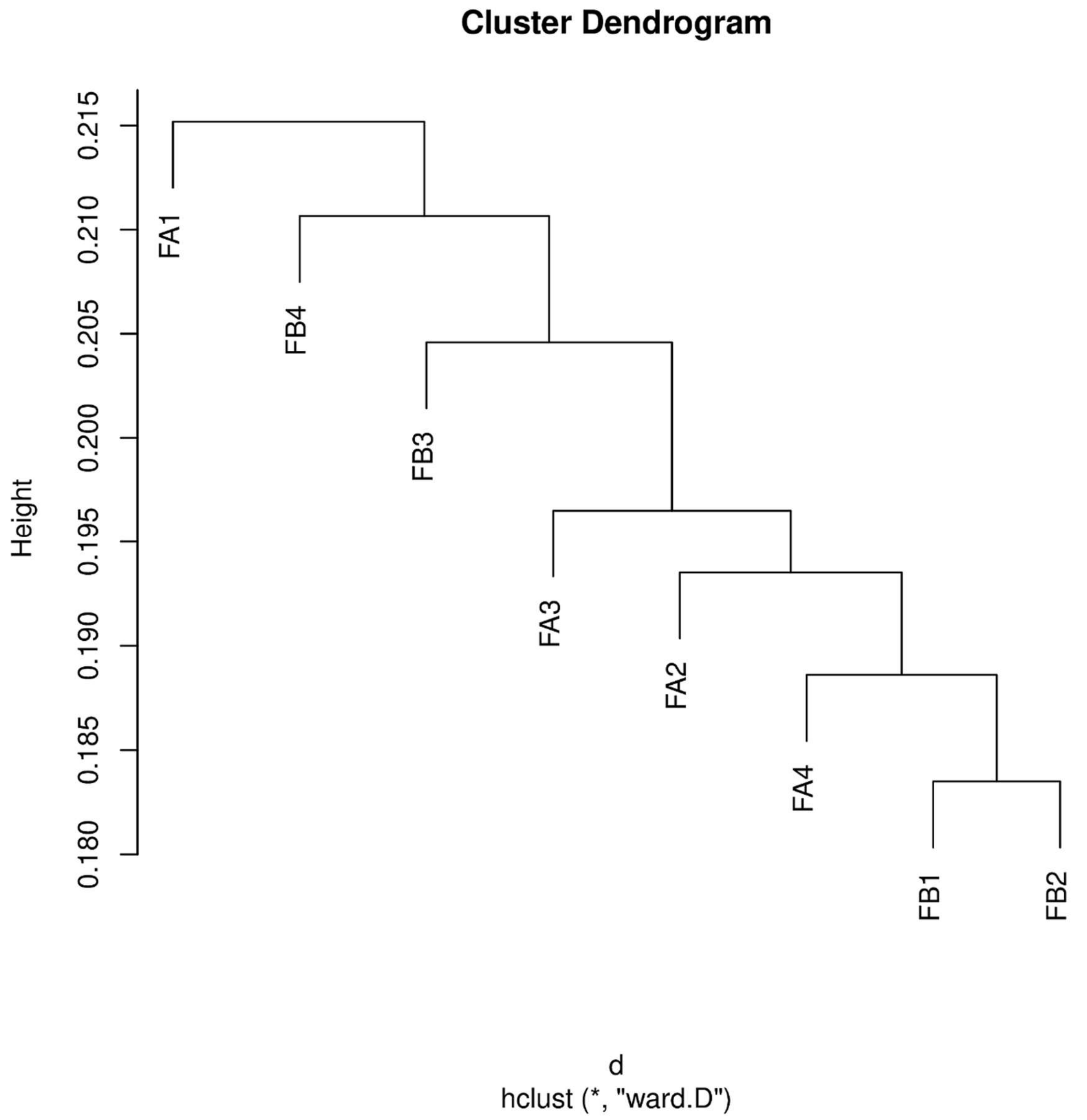

Figure S1.7. Hierarchical Cluster Dendrogram
FB samples indicate poly(I:C) samples (n=4), FA samples indicate vehicle samples (n=4). The dendrogram visually represents sample distance by hierarchical clustering. Height represents the similarity between individual samples..

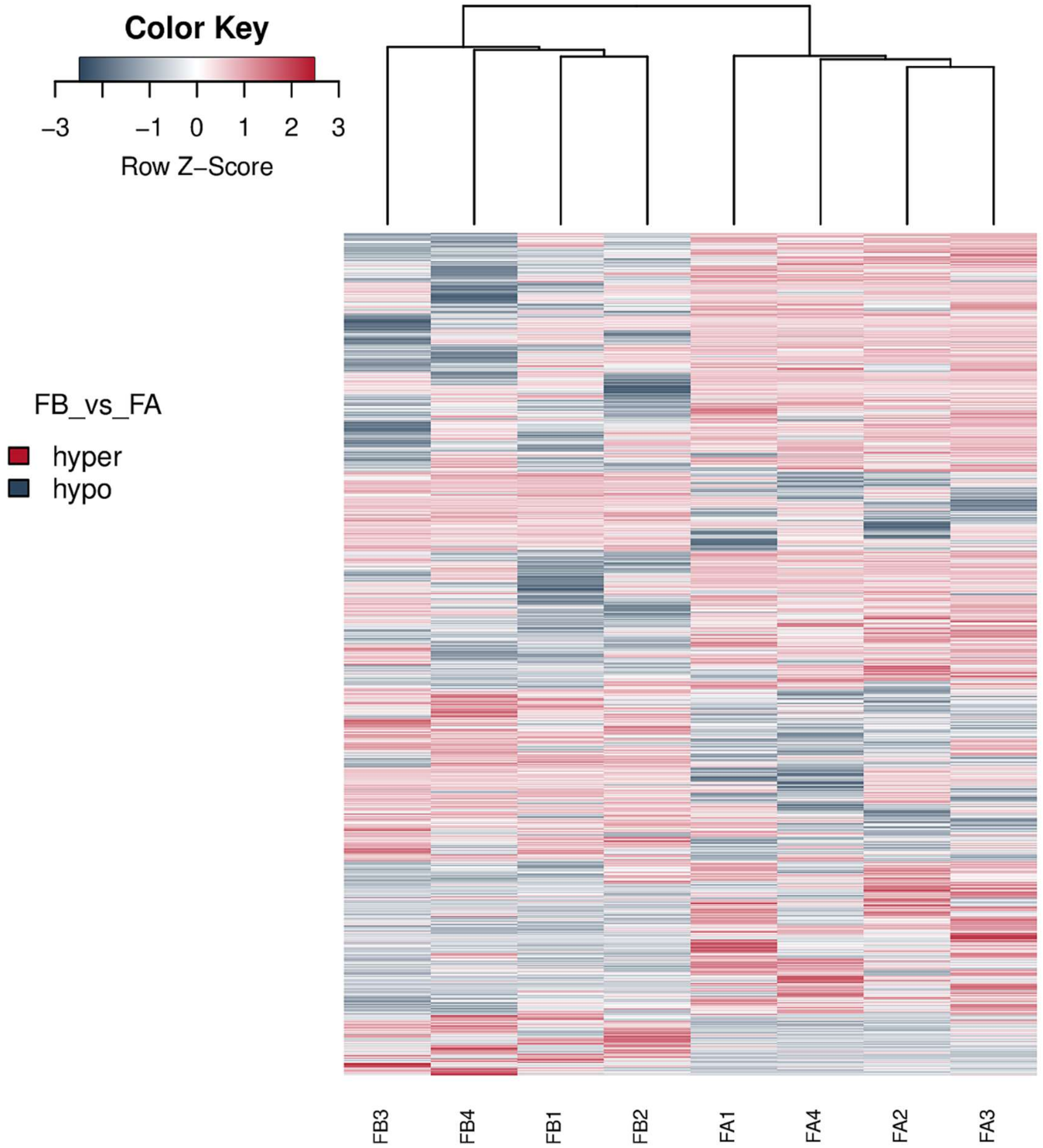

Figure S1.8. Hierarchical Cluster Dendrogram
The main branches in this type of dendrogram show how the two clusters of samples (Poly(I:C) and vehicle) are distinct from each other, and how the samples within each cluster are broadly similar to each other. The heatmap is used to visualize the overall distribution of differential methylation between the groups based on whether a DMR is hypomethylated or hypermethylated. The distance in percentage methylation from the mean is obtained as z-scores. A positive z-score means that the DMR methylation percentage was above the average across samples, whereas a negative one means it was below average. FB samples indicate poly(I:C) samples (n=4), FA samples indicate vehicle samples (n=4).

Supplement S3. Chromosomal location of all RRBS-identified DMCs and DMRs.
This is a separate supplement and comprises the following Supplementary Tables:

Table S3.1. Unfiltered DMCs. Table denotes all DMCs (Poly(I:C) vs Vehicle) with their chromosomal and strand position.

Table S3.2. Filtered DMCs. Table denotes DMCs (Poly(I:C) vs Vehicle) which passed the cut off criteria (qvalue≤0.01, meth.diff ±25%) with their chromosomal and strand position.

Table S3.3. Unfiltered DMRs. Table denotes all DMRs (Poly(I:C) vs Vehicle) with their chromosomal and strand position.

Table S3.4. Filtered DMRs. Table denotes DMRs (Poly(I:C) vs Vehicle) which passed the cut off criteria (qvalue≤0.01, meth.diff ±25%) with their chromosomal and strand position.

S2.3. Genomic annotations of identified DMCs and DMRs

All DMCs and DMRs were annotated to genomic location within the rat reference genome Rn5.0. It was found that both DMCs and DMRs were evenly distributed across all chromosomes (Figure S1.9) with percentage change in gene methylation between groups ranging from -78.4% to +85.9% for DMCs, and -75.7% to +75.9% for DMRs (Supplement S4).

CpG context mapping showed the majority of DMCs (Table S4.1) and DMRs (Table S4.2) were mapped to open sea regions, 76.9% and 85.3%, respectively. For DMRs, the fewest proportion were mapped to CGIs (1.5%) with identical numbers mapped to shelves and shores (6.6%). By comparison, for DMCs, the second highest mapping was to CGIs (9.8%), then shores (9.1%) with shelves having the least DMCs (4.3%).

Gene context mapping showed that the majority of DMCs (Table S5.2) and DMRs (Table S5.4) mapped to intergenic, 55.3% and 53.2%, respectively, followed by introns (29.0% and 33.4% respectively), then exons (12.6% and 11.4% respectively), and with the least number mapped to promoters (3.1% and 2.1% respectively). Gene mappings, including promoters, exons and introns are shown Table S5.1 and S5.3 for DMCs and DMRs, respectively. These genic regions were used to interpret the differentially methylated genes, used for downstream functional analysis. Intergenic region mappings are shown in Table S5.2 and S5.4 for DMCs and DMRs, respectively.

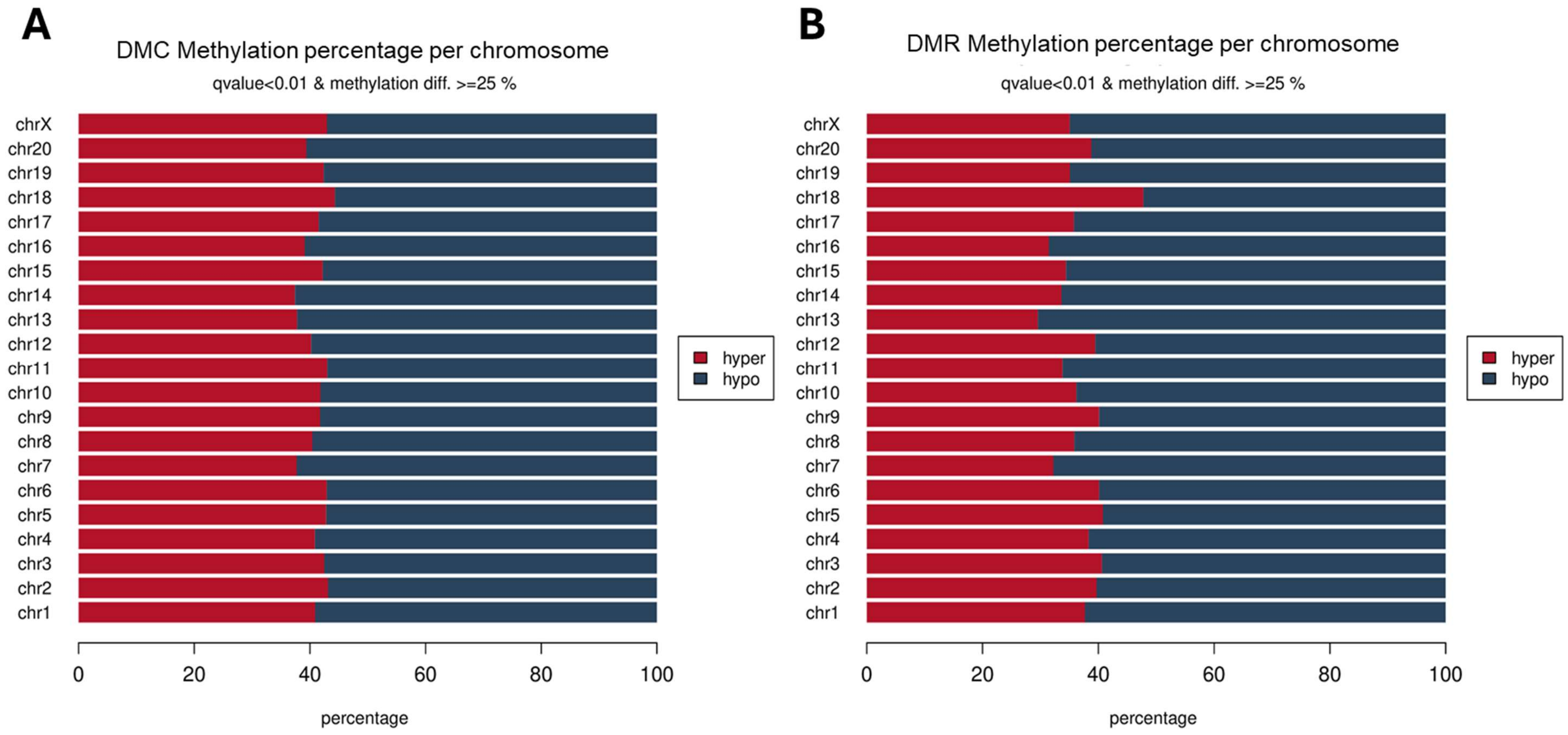

Figure S1.9. Chromosomal mapping of differentially methylated CpG sites (DMC) and regions (DMR).
Figures show the percentage of hypomethylation (blue) and hypermethylation (red) with respect to **A.** DMCs and **B.** DMRs within each chromosome in the rat genomes.

Supplement S4. CpG context mapping of filtered DMCs and DMRs.
This is a separate supplement and comprises the following supplementary Tables:

Table S4.1. CpG context mapping of filtered DMCs. Table denotes the CpG context of all filtered DMCs (Poly(I:C) vs Vehicle) including CGI, CpG shore (2000bp flanking region of a CGI), CpG Shelve (within 2000bp of the CpG shore), Open Sea (intergenic)

Table S4.2. CpG context mapping of filtered DMRs. Table denotes the CpG context of all filtered DMRs (Poly(I:C) vs Vehicle) including CGI, CpG shore (2000bp flanking region of a CGI), CpG Shelve (within 2000bp of the CpG shore), Open Sea (intergenic)

Supplement S5. Gene annotation of filtered DMCs and DMRs.
This is a separate supplement and comprises the following supplementary Tables:

Table S5.1. Gene annotation of filtered DMCs. Table denotes gene annotations of all filtered DMCs (Poly(I:C) vs Vehicle) based on their mapping to exons, introns and promoters.

Table S5.2. Intergenic annotation of filtered DMCs. Table denotes intergenic annotations of all filtered DMCs (Poly(I:C) vs Vehicle). These DMCs could not be annotated to genes based on their mapping to intergenic regions.

Table S5.3. Gene annotation of filtered DMRs. Table denotes gene annotations of all filtered DMRs (Poly(I:C) vs Vehicle) based on their mapping to exons, introns and promoters.

Table S5.4. Intergenic annotation of filtered DMRs. Table denotes intergenic annotations of all filtered DMRs (Poly(I:C) vs Vehicle). These DMRs could not be annotated to genes based on their mapping to intergenic regions.

S2.4. Analysis of MIA-induced differentially methylated genes

We then performed Gene Ontology analysis on the gene set inclusive of biological process (Table S6.1), molecular function (Table S6.2) and cell component (Table S6.3). The top 20 biological processes demonstrated an enrichment for functions in neuronal development, including synapse/axon generation and cell differentiation. Of note, there was also a significant enrichment for genes involved in behaviours relevant to schizophrenia, including locomotor behaviour, memory, learning and cognition. For molecular functions, many of the top 20 were associated with transmembrane transport/signalling, notably those involved with ion transport by transmembrane channels. There was also DNA-binding transcription factor activity, actin binding, kinase activity and cell adhesion. The top 20 cell components were primarily enriched for synaptic membranes, and cell-cell junctions and main/distal axon functions. Taken together, the Gene Ontology assessments support an enrichment of the differentially methylated genes in neurodevelopmental processes and neuronal signalling, both implicated in the pathogenesis of neurodevelopmental disorders such as schizophrenia.

The RRBS gene list was assessed via KEGG pathway analysis (Table S6.4). In support of the Gene Ontology findings, the KEGG pathway analysis showed that the top ten enriched pathways were those involved in normal neuronal functional and neuronal signalling pathways, and therefore further support disturbed neurodevelopmental processes.

Supplement S6. Gene Ontology and KEGG pathway analysis.
This is a separate supplement and comprises the following supplementary Tables:

Table S6.1. Gene Ontology: Biological processes. Table lists the differentially methylated genes significantly mapped to biological processes in the gene ontology database.

Table S6.2. Gene Ontology: Molecular function. Table lists the differentially methylated genes significantly mapped to molecular functions in the gene ontology database.

Table S6.3. Gene Ontology: Cell component. Table lists the differentially methylated genes significantly mapped to cell components in the gene ontology database.

Table S6.4. KEGG pathways. Table lists the differentially methylated genes significantly mapped to KEGG pathways.
