# Supplementary material for: Developmental modulation of schizophrenia risk gene methylation in offspring exhibiting cognitive deficits following maternal immune activation
Source: Mol Psychiatry. 2025 Aug 29;31(1):418–29. doi: 10.1038/s41380-025-03147-1 (PMC12700827; doi:10.1038/s41380-025-03147-1)
Supplement: Supplementary file 3 — Supplement S2: Maternal Immune Activation (MIA) Model Reporting Guidelines Checklist. [file 41380_2025_3147_MOESM3_ESM.pdf]

Table S1

Maternal Immune Activation Model Reporting Guidelines Checklist

ARRIVE Reporting Guideline & Recommendation	Arrive Item	MIA Model Specific Reporting Recommendation *Please complete this chart for each point outlined below. If not applicable, write N/A*
**Study design**<br>➢Overview of immune activation issues<br><br>For each experiment, give brief details of the study design including:<br>a. The number of experimental and control groups.<br>b. Any steps taken to minimize the effects of subjective bias when allocating animals to treatment (e.g. randomization procedure) and when assessing results (e.g. if done, describe who was blinded and when).<br>c. The experimental unit (e.g. a single animal, group or cage of animals).<br><br>A time-line diagram or flow chart can be useful to illustrate how complex study designs were carried out.	6	MIA Specific Reporting:<br>a. General need for improved reporting in MIA model methods + reporting pilot data<br>○ Details on pilot data:<br><br>Experimental procedures were based on previous studies within the group that have systematically investigated the appropriate dose and strain, and commercial source of poly(I:C) with respect to the biomolecular properties of molecular weight and negligible endotoxin contamination (16, 17,19).<br>Animals used in this study were those detailed in two of our previously published studies (17,19).
**Experimental procedures**<br>➢Compounds<br>➢Validation measures<br><br>For each experiment and each experimental group, including controls, provide precise details of all procedures carried out. For example:<br>a. How (e.g. drug formulation and dose, site and route of administration, anaesthesia and analgesia used [including monitoring], surgical procedure, method of euthanasia). Provide details of any specialist equipment used, including supplier(s).<br>b. When (e.g. time of day).<br>c. Where (e.g. home cage, laboratory, water maze).<br>d. Why (e.g. rationale for choice of specific anaesthetic, route of administration, drug dose used).	7	Provide details of:<br>a. Compounds – source, vehicle, preparation/storage, administration route, volume administered, whether anesthetics were used at time of immune challenge.<br>○ Name of compound: Polyinosinic:polycytidylic acid (poly(I:C)) (InvivoGen)<br>○ Catalogue number: tlrl-picw<br>○ Lot number: PIW-38-04<br>○ Vehicle control used: Endotoxin-free 0.9 % saline<br>○ Route of administration: Intraperitoneal<br>○ Volume administered: 10 mg/kg bodyweight<br>○ Storage conditions: -20°C as 500 µL aliquots. Max. 1 freeze/thaw cycle<br>○ Anesthetic (type, dose, duration) used:<br>None<br>b. Housing variables at injection - temperature of room at injection time, cage change at time of injection or not<br>○ Light cycle of animal housing room: 12h:12h, lights on at 07.00<br>○ Time of day of injection: 09.00-11.00<br>○ Room temperature at injection time: 21± 2 °C<br>○ Did a cage change occur at time of injection: NO

		c. Validation of immune activation – behavior, physiological indices and/or cytokine data, including pilot dosing data<br>○ Method used to verify immune activation:<br>Maternal plasma was taken from the lateral tail vein at 3 hours post-treatment. This time was selected as we have previously validated this to correspond to the peak of the maternal inflammatory response to poly(I:C) (16). Commercially available ELISAs for rat IL-6 and TNFα were used to confirm the maternal inflammatory response (see Figure S1.2). Maternal weight change was also measured throughout gestation.<br>d. Validation of gestational timing – vaginal plug, estrous cycle, weight gain<br>○ Method of validating gestational timing:<br>Rats were time mated with one male and one female per cage. Mating cages were checked twice daily (09.00 hrs and 17.00 hrs) for the presence of a vaginal plug. The presence of a plug was designated gestational day (GD) 1. Weight was measured daily at 17.00 hrs. All dams gave birth between GD22-23.<br><br>Additional comments:<br>Commercial source of poly(I:C) was chosen based on a previous validation study showing low variability of molecular weight and endotoxin contamination compared to the leading supplier (16).
**Experimental animals**<br>➢Species/strain/vendor<br><br>a. Provide details of the animals used, including species, strain, sex, developmental stage (e.g. mean or median age plus age range) and weight (e.g. mean or median weight plus weight range).<br>b. Provide further relevant information such as the source of animals, international strain nomenclature, genetic modification status (e.g. knock-out or transgenic), genotype, health/immune status, drug or test naïve, previous procedures, etc.	8	Provide details of:<br>a. Species – considerations for appropriate species (mouse, rat, non human primate, other)<br>○ Species: Rat<br><br>b. Strain – variability in strain can influence model<br>○ Strain: Wistar<br><br>c. Maternal/Offspring Physiological Variables at time of immune challenge – age, body weight<br>○ Maternal Age at challenge: 8-12 weeks old, treatment on GD15<br>○ Maternal Body weight: 322.5 ± 3.61 g (mean ± SEM) on GD15<br>○ Offspring Age at challenge: N/A<br>○ Offspring Sex: Both males and females tested<br>○ Offspring Body weight: Measured weekly from birth<br><br>d. Vendor – even within the same strain, vendor can influence endpoints<br>○ Vendor: Charles River<br>○ Location of Vendor: UK<br>○ Room/area where animals originated from: Not known

		Additional Comments:<br>Dams had a baseline (pre-pregnancy) weight of 260.6 ± 2.70 g (mean ± SEM).
**Housing and husbandry**<br>➢Cage, ventilation, bedding, enrichment<br><br>Provide details of:<br>a. Housing (type of facility e.g. specific pathogen free [SPF]; type of cage or housing; bedding material; number of cage companions; tank shape and material etc. for fish).<br>b. Husbandry conditions (e.g. breeding program, light/dark cycle, temperature, quality of water etc for fish, type of food, access to food and water, environmental enrichment).<br>c. Welfare-related assessments and interventions that were carried out prior to, during, or after the experiment.	9	Provide details of:<br>a. Caging systems<br>○ *At breeding*<br>Material of cage: Plexiglass<br>Cage dimensions: 1934 mm x 888 mm x 1986 mm<br>○ *After parturition*<br>Material of cage: Plexiglass<br>Cage dimensions: 1934 mm x 888 mm x 1986 mm<br>○ *At weaning*<br>Material of cage: Plexiglass<br>Cage dimensions: 1934 mm x 888 mm x 1986 mm<br><br>b. Animal Holding room<br>○ Temperature in room: 21 ± 2 °C<br>○ Humidity in room: 55 ± 5 %<br>○ Ventilation system: Individually-ventilated cages<br>○ Specific pathogen free [SPF]: NO<br>○ Are males & females housed in the same or separate rooms: Housed in same room after weaning<br>c. Bedding exchanges/bedding type<br>○ Type of cage bedding used: Sawdust and sizzle nest<br>○ Frequency of cage changes per week<br>*during gestation:* 1<br>*during neonatal period:* 1<br>*following weaning:* 1<br><br>d. Breeding - bred on site or timed pregnant, how many different sires (are the same fathers breeding with both experimental and control dams)<br>Breeding location: On site

		○ Gestational age at shipping: N/A (not pregnant)<br>○ Biological age of dams (if not listed in Section 8c): 8-12 weeks<br>○ Number of Dams bred: 30<br>○ How many times have dams been mated previously: 0<br>○ How many times did the dams mate and not become pregnant: 4<br>○ Are the dams primiparous or multiparous? All dams are primiparous<br>○ What was the frequency of maternal handling during the gestational/neonatal period (e.g. cage cleanings, weighing, blood collection manipulations): Weighed once/day; cleaned once/week<br>○ Biological age of sires: 8-12 weeks<br>○ Number of sires bred: 10<br>○ How many times have sires been mated previously: None<br>○ How many times did the sires mate successfully (e.g. mating resulted in pregnancy, full term birth): 30/34 (88% success)<br>○ If bred previously, what was the interval between mating times:<br>○ Are sires matched to experimental and control dams: NO<br>○ Describe the mating design (1:1, 1:2 etc): 1:1<br><br>e. Social enrichment – number of cage companions<br>○ Number of cage companions prior to breeding: 5 females/cage<br>○ Gestational age when dam separated for parturition: 17 (21 for fetal<br>○ Number of cage companions at weaning: 5 females or 3 males/cage<br><br>f. Physical enrichment – describe enrichment devices, and when enrichment is in the cage (removed when pups born? Or present throughout study), does the enrichment type change? How frequently?<br>○ Describe what type of enrichment devices (and how many) are included in cage/housing room:<br>Split-level enrichment in individually ventilated cages (IVCs) (GR1800 Double-Decker Cage, Techniplast, UK; catalogue number 2GR16DD), cardboard tube with sizzle nest bedding, wooden chew toy(s).

		○ Does enrichment type/access change across study? NO<br>○ If so, when does enrichment type/access change (e.g. enrichment removed prior to parturition and replaced in late neonatal period):<br>NA<br><br>Additional Comments:<br>NA
**Sample size**<br>➢Litter versus offspring<br><br>a. Specify the total number of animals used in each experiment, and the number of animals in each experimental group.<br>b. Explain how the number of animals was arrived at. Provide details of any sample size calculation used.<br>c. Indicate the number of independent replications of each experiment, if relevant.	10	Provide details of:<br>a. Maternal N vs offspring N<br>○ What is the total number of dams/litters included in the study: 29<br>○ What is the total number of offspring per litter included the study: 10-12/litter for postnatal samples (maximum 2 of the same sex at each<br>b. Litter size and sex distribution<br>○ What size was each litter maintained at: max 10<br>○ What age did culling take place at: Postnatal day (PD) 1<br>○ How many males and females were maintained in each litter: 5 males/5 females where possible (see Additional Comments)<br>c. Cross fostering<br>○ Did cross fostering occur: NO<br>○ If so, at what age did cross fostering occur: NA<br><br>Additional Comments:<br>Dams assigned to prenatal culls where fetal harvests were performed the whole litter was taken for downstream analysis at a single tiempoint. For Dams assigned to postnatal litters, where possible litters were culled to 5 males and 5 females on PD1. For litters that had fewer for one sex, total litter size was maintained at 10 pups to reduce the effect on maternal behaviour (e.g. a litter of 3 males and 10 females would be culled to 3 males and 7 females). For litters below 10 pups, none were culled on PD1.

**Allocating animals to experimental groups**<br><br>a. Give full details of how animals were allocated to experimental groups, including randomization or matching if done.<br>b. Describe the order in which the animals in the different experimental groups were treated and assessed.	11	a. How many offspring per litter were used in each measure: Maximum 4 (2 males and 2<br><br>b. Randomization/Matching procedures<br>○ What procedures were used to assign animals to groups:<br>Randomised to treatment group, to which the experimenters were blinded.<br><br>c. Sex as a biological variable (behavioral and physiological outcomes)<br>○ Were both males and females evaluated in each behavioral and physiological outcome: YES<br><br>Additional Comments:<br>Both males and females were included in all analyses. At each timepoint in the study, a maximum of two fetuses/pups of the same sex were used from a single litter.
**Experimental outcomes**<br>➢ Behavioral testing<br>➢ Physiological endpoints<br><br>Clearly define the primary and secondary experimental outcomes assessed (e.g. cell death, molecular markers, behavioral changes).	12	a. Maternal behavior and pup interactions<br>○ If maternal care was evaluated, were there differences following immunogen challenge (if so, please briefly describe):<br>Details on specific maternal care behaviours relating to the animals in this study can be found in our previous study (19).<br><br>b. Age(s) of offspring at behavioral testing/physiological evaluation endpoints:<br>Molecular endpoints included prenatal (fetal) samples collected at GD21, and postnatal offspring samples collected from neonates (PD1), infants (PD21) adolescence (PD35) and adulthood (PD175).<br>Details on specific offspring behaviours relating to the animals in this study can be found in our<br>c. Order of testing (e.g. behavioral test order)<br>○ Were animals evaluated in a counter-balanced order in terms of:<br>*presentation of tests to each animal:* NO<br>*order of experimental/control groups run through each test:* YES<br>○ What was the inter-test interval if a single animal underwent a battery of tests:<br>1-2 days

		Additional Comments:<br>Details on sample collection can be found in methods. Details on bheavioural testing can be found in our previous publication (19).
**Statistical methods**<br><br>a. Provide details of the statistical methods used for each analysis.<br>b. Specify the unit of analysis for each dataset (e.g. single animal, group of animals, single neuron).<br>c. Describe any methods used to assess whether the data met the assumptions of the statistical approach.	13	a. Unit of analysis for each data set<br>○ Is the unit (n) of each analysis based on number of litters, or number of animals used per group:<br>For fetal and offspring data, each data point represented a single pup with Dam ID used as a random factor in all analyses to account for variability based on shared maternal environment(s).
**Other Disclosures**		Please make note of any other extraneous variables that you would like to report (e.g. fire alarms, construction, temporary relocations, other variables that you think we should be considering in our studies etc.):<br>NA

The recommended use of this reporting form is to fill it out and include it as supplemental material for each of your laboratory's research publications. If there are difficulties utilizing/adapting this fillable form, please contact one of the corresponding authors to request a copy. The authors give permission for this table to be edited for use in reporting on other animal models (e.g. postnatal immune challenge models, early life stress models) as appropriate.

Kentner AC, Bilbo AD, Brown AS, Hsiao EY, McAllister AK, Meyer U, Pearce BD, Pletnikov MV, Yolken RH, Bauman MD. (2018). Maternal immune activation: reporting guidelines to improve the rigor, reproducibility, and transparency of the model. Neuropsychopharmacology, https://doi.org/10.1038/s41386-018-0185-7.
